# CRAFTing Delivery of Membrane Proteins into Protocells using Nanodiscs

**DOI:** 10.1021/acsami.3c11894

**Published:** 2023-11-28

**Authors:** Piotr Stępień, Sylwia Świątek, Manuel Yamil Yusef Robles, Joanna Markiewicz-Mizera, Dhanasekaran Balakrishnan, Satomi Inaba-Inoue, Alex H. De Vries, Konstantinos Beis, Siewert J. Marrink, Jonathan G. Heddle

**Affiliations:** † Malopolska Centre of Biotechnology, Jagiellonian University, Krakow 30-387, Poland; ‡ Postgraduate School of Molecular Medicine, Żwirki i Wigury 61, Warsaw 02-091, Poland; § Department of Life Sciences, Imperial College London, Exhibition Road, South Kensington, London SW7 2AZ, U.K.; ∥ Rutherford Appleton Laboratory, Research Complex at Harwell, Didcot, Oxfordshire OX11 0FA, U.K.; ⊥ Groningen Biomolecular Sciences and Biotechnology Institute, University of Groningen, Groningen 9747 AG, The Netherlands

**Keywords:** nanodiscs, liposomes, fusion, membrane protein delivery, synthetic biology

## Abstract

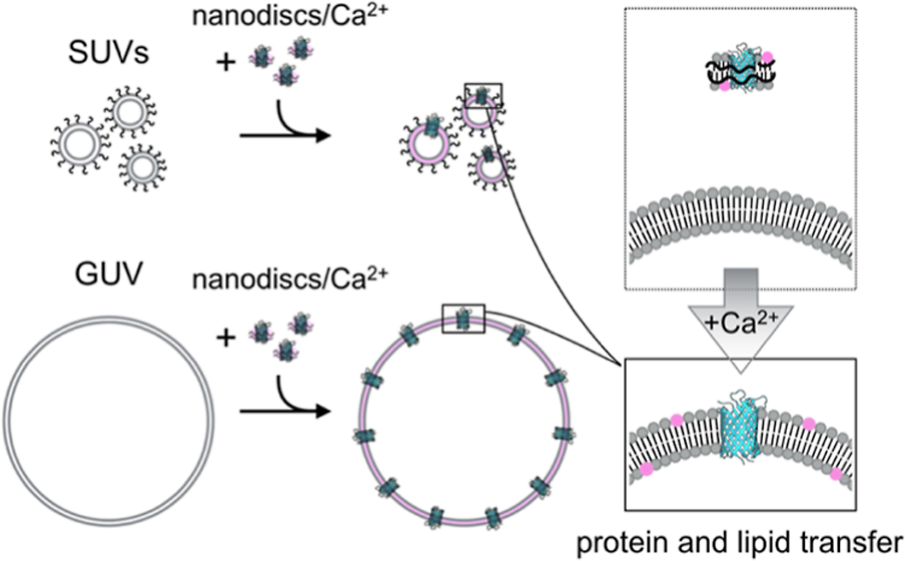

For the successful generative engineering of functional artificial cells, a convenient and controllable means of delivering membrane proteins into membrane lipid bilayers is necessary. Here we report a delivery system that achieves this by employing membrane protein-carrying nanodiscs and the calcium-dependent fusion of phosphatidylserine lipid membranes. We show that lipid nanodiscs can fuse a transported lipid bilayer with the lipid bilayers of small unilamellar vesicles (SUVs) or giant unilamellar vesicles (GUVs) while avoiding recipient vesicles aggregation. This is triggered by a simple, transient increase in calcium concentration, which results in efficient and rapid fusion in a one-pot reaction. Furthermore, nanodiscs can be loaded with membrane proteins that can be delivered into target SUV or GUV membranes in a detergent-independent fashion while retaining their functionality. Nanodiscs have a proven ability to carry a wide range of membrane proteins, control their oligomeric state, and are highly adaptable. Given this, our approach may be the basis for the development of useful tools that will allow bespoke delivery of membrane proteins to protocells, equipping them with the cell-like ability to exchange material across outer/subcellular membranes.

## Introduction

A major goal in synthetic biology is the bottom-up construction of artificial cells. As per the definition proposed by Jeong et al.,^1^ these are assembled from cellular molecules (e.g., phospholipids, proteins, etc.) and able to produce energy, at least some of which is used for their own metabolic activities. Also termed “typical artificial cells” by Jiang et al., they are analogous to natural cells in terms of structure and capabilities.^2^ Consequently, to ensure native-like functionality, several structures are required. Crucial among these is a (lipid) membrane acting as a physical and thermodynamic barrier separating the cell from the external environment. Smaller, membrane-bound compartments in the artificial cell interiors are necessary to perform specific tasks, which may need to be localized.

For cells to function, information and materials must be exchanged across both the external cell and internal compartmental membranes. This role is largely carried out by membrane-spanning proteins^3^ and similar structures will be required by artificial cells. While synthetic production of lipid bilayer “containers,”, that is, GUVs is trivial, equipping both the external membrane and the membranes of internal compartments with desired proteins is challenging. Most commonly used strategies capitalize on the fact that membrane proteins can be readily incorporated during GUV preparation.^4^ However, there are numerous issues associated with this coassembly approach including incorrect orientation of proteins.^5^ This can be overcome by using charged lipids in the GUV that carry an opposite charge to that on the protein.^6^ However, this may lead to a non-natural lipid membrane composition, with the requirement for a significant opposing charge on the membrane protein being a further limitation. More importantly, coassembly or the more advanced detergent-assisted insertion^7^ is not suitable when several different integral membrane proteins (necessary for full functionality) are required due to their differing detergent compatibility. The choice of detergent for extraction and purification still remains a major bottleneck in membrane protein research^8,9^ which only increases as the diversity of proteins increases in more sophisticated systems. One solution with the potential to overcome all of these problems is to place the molecular machinery required for in-cell production and translocation of membrane proteins into the liposome such that native-like in situ production and placement is achieved.^10,11^ However, this introduces a high degree of complexity into the system. A simpler and more scalable solution would be easier to integrate into artificial and engineering biology procedures.

The alternative approach that mitigates some of these problems is the delivery of membrane proteins into preformed GUVs without the use of detergents, which mimics delivery of membrane proteins from endoplasmic reticulum to other organelles. This, in principle, should allow for more block-by-block construction of artificial cells. To this end, SUV–GUV fusion has been employed based on charge complementarity of negatively charged GUVs and positively charged SUVs. In this way a functional complex of membrane proteins can be reconstituted.^12,13^ However, the main drawback of this method is the use of non-naturally occurring positively charged lipids, which in turn limits applicability. Another approach for preparation of semisynthetic protocells was proposed which uses calcium driven fusion of cell-derived plasma membrane vesicles with phosphatidylcholine enriched GUVs.^14^ This method, while providing a completely native membrane environment and omitting detergent, requires an elaborate microfluidic setup, which limits SUVs and GUVs cross-fusion. Moreover, within this technique, complete control of protein composition is not possible, thus compromising the ideal of a finely controlled, bottom-up, artificial cell construction. Given the shortcomings of currently available detergent-free solutions, we decided to develop an alternative strategy for delivery of membrane protein to preformed vesicles which would utilize naturally occurring lipids, be scalable, be rapid to deploy, and be easily integrated into more complex systems. Additionally, the carrier molecules should be small enough to be able to carry individual membrane proteins; they should be stable and possess potential for further modifications allowing them to interface with other tools available in synthetic biology.

In this report, we propose and demonstrate this novel approach for delivering membrane proteins to preformed liposomes, by developing a nanodisc-based Calcium Responsive Artificial Fusion Transfer system (nano-CRAFT), which addresses most major disadvantages of current alternative approaches. It uses naturally occurring negatively charged phosphatidylserine membranes, providing native-like conditions for the proteins in pre- and postfusion membranes. The protein delivery is detergent independent, is achieved in a simple one-pot reaction, and does not require microfluidics system. Additionally, nano-CRAFT can be applied to both preformed GUVs and SUVs, making it a powerful addition to the synthetic biology toolbox capable of embedding lipid and membrane protein components into large or small membrane bound compartments, giving it potential use as synthetic cell building blocks for cell-membrane and organelles, respectively.

## Results and Discussion

Nanodiscs, first developed by Sligar et al.,^15^ are nanoscale patches of lipid bilayer stabilized by two antiparallel belts of membrane scaffold protein (MSP). Nanodisc diameter can be controlled via modification of the MSPs and their lipid contents are fully addressable. They can be used as a platform for handling monomeric and oligomeric membrane proteins and complexes thereof.^16^ In contrast to similar peptide^17^ and polymer-based discs,^18^ MSP-based nanodiscs do not appear to undergo extensive interparticle lipid transfer,^19^ making them the best choice for use as a stable carrier particle.

Nanodisc–liposome fusion has been previously studied to gain insight into synaptic processes, to understand the fusion pore,^20^ and for its potential in drug delivery.^21,22^ However, the first approach is based on utilizations of elaborate fusion machinery, and the second requires hours for significant fusion to occur. We aimed to find a simpler, rapid, and scalable method more suited for future application in in vitro artificial cell production. To this end we decided to use calcium driven fusion, a well-studied process,^23−25^ in which two phosphatidylserine containing lipid bilayers, (e.g., liposomes) can coalesce after calcium addition. Calcium allows the membranes to overcome repulsion of negatively charged phosphatidylserine (PS), and subsequently dehydrates the membranes and induces a negative curvature,^26^ leading to prompt membrane fusion.

**Figure 1:**
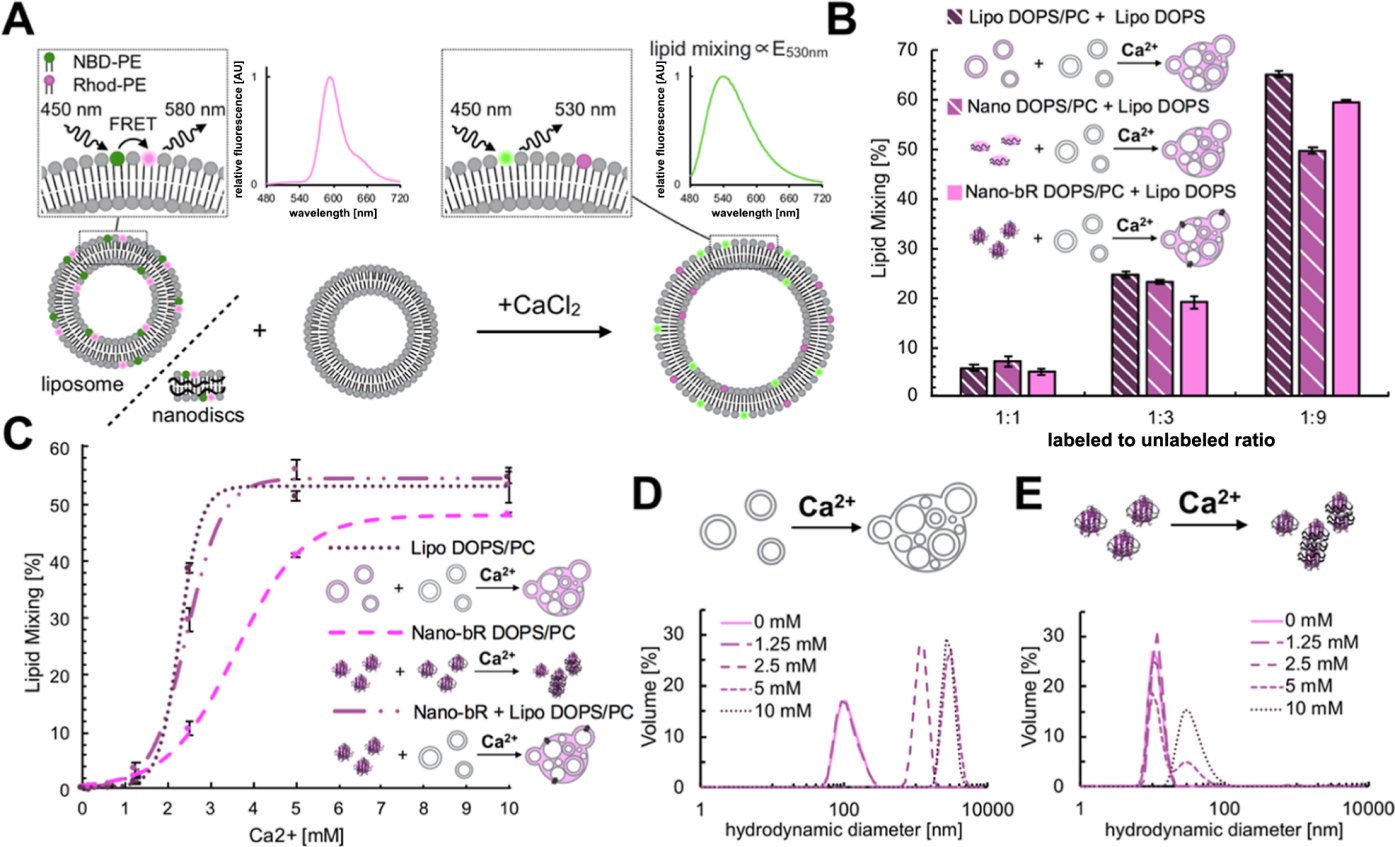
(A) Scheme of lipid mixing detection: initially, upon 450-nm excitation, only the acceptor (Rhod-PE) fluorescence can be observed. After fusion, the increase in surface area of the resulting product vesicles separates the NBD-PE and Rhod-PE FRET pair, leading to recovery of donor (NBD-PE) fluorescence at 530 nm which is proportional to lipid mixing. (B) Comparison of lipid mixing between the fusion of SUV–SUV liposomes and fusion between MSP1E3D1 nanodisc (with and without bR) and SUVs. The experimental setup with expected product is illustrated schematically along with the ratio between the colored labeled population (DOPS/Rhod-PE/NDB-PE; 96:2:2) and unlabeled population (DOPS). (C) Measurements of cross-lipid mixing between nanodiscs-bR and SUV liposomes compared to nanodiscs-bR liposome fusion for different concentrations of Ca^2+^. The colored labeled population (DOPS/DOPC/Rhod-PE/NDB-PE; 83:13:2:2) and unlabeled population (DOPS/DOPC; 85:15) were mixed at 1:9 ratio. The curves were fitted using the equation *F*(*x*) = *A*/(1 + exp(−*Bx* + *C*). The effect of Ca^2+^ on particle size distribution (hydrodynamic diameter) of liposomes (D) and nanodisc-bR (E) as measured by DLS for the DOPS/DOPC/Rhod-PE/NDB-PE (85:13:2:2) lipid composition.

First, we asked if the two lipid bilayers to be fused in this process could be provided by an SUV and a nanodisc. In order to answer this question we prepared “delivery” DOPS/DOPC (1,2-dioleoyl-*sn*-glycero-3-phospho-l-serine/1,2-dioleoyl-*sn*-glycero-3-phosphocholine Table S1) and MSP1E3D1 based nanodiscs with and without inclusion of bacteriorhodopsin (bR) as a model membrane protein and tested their fusion with “target,” purely DOPS based ∼100 nm diameter SUV liposomes using a well-established lipid mixing assay.^25^ In this assay the nanodiscs (or control SUV liposomes) are prepared containing fluorescently labeled lipids, 2 mol % of NBD-PE (1,2-dioleoyl-*sn*-glycero-3-phosphoethanolamine-*N*-(7-nitro-2-1,3-benzoxadiazol-4-yl), and 2 mol % of Rhod-PE (Rhod-PE, 1,2-dioleoyl-*sn*-glycero-3-phosphoethanolamine-*N*-(lissamine rhodamine B sulfonyl), which together constitute a FRET pair, and are subsequently mixed with increasing amounts of unlabeled SUVs. If fusion occurs, the FRET pair from the “delivery” nanodiscs of liposome is diluted into the larger pool, increasing the average distance between the two, which is measured as the increase of donor NBD-PE fluorescence (Figures 1A and S1). The base lipid composition of “delivery” liposomes and nanodiscs was set to 75:25 mol/mol DOPS/DOPC due to suboptimal yields of incorporation of bR into pure DOPS. The addition of PC at this concentration was previously reported not to abolish Ca^2+^ driven fusion.^27,28^ We have specifically chosen MSP1E3D1-based nanodiscs with a diameter of ∼12.8 nm^29^ as ∼6.5 nm^2^ monomeric bR would take up only ∼7% of the membrane surface leaving the remainder readily fusogenic.

Upon addition of a standard^25^ fusion-triggering CaCl_2_ concentration (5 mM) lipid transfer from nanodiscs to liposomes was observed with the measured lipid mixing between both types of nanodiscs and liposomes being comparable to that seen in the control liposome–liposome system (Figure 1B). This shows that neither the presence of MSP1E3D1, which stabilizes the lipid bilayer, nor the presence of bR strongly limits the calcium driven lipid delivery from nanodiscs to target vesicles. The fusion efficiency also increases as the relative concentration of unlabeled receiving vesicles increases, indicating that the lipids transferred from the nanodiscs are not trapped after a single round of fusion and can be further diluted by unlabeled liposome–liposome fusion.

This experimental setup is limited in two significant ways (i) it does not provide information on the undesired nanodiscs–nanodiscs fusion/aggregation processes; (ii) while undetectable in the lipid mixing assay, the fusion of unlabeled liposomes occurs, resulting in uncontrollable aggregation^30^ of the system, rendering it unsuitable for downstream applications (Figure 1D).

To ensure the applicability of the studied system for delivery of membrane proteins, cross-interactions of the nanodisc particles carrying bR were first investigated to assess possible nanodisc–nanodisc self-aggregation. For this, we compared the calcium driven interactions between nanodiscs to those between SUVs and to fusion of nanodiscs with SUVs, where all the systems shared our “delivery” lipid composition (DOPS/DOPC; 75:25, Table S1). To this end lipid mixing was measured using a 1:9 lipid ratio of labeled/unlabeled molecules for different triggering calcium concentrations. The same conditions were also probed using DLS (dynamic light scattering) measurements to track particle size changes and possible aggregation.

Liposome cross-fusion is triggered by 2.5 mM Ca^2+^ as reported by lipid mixing and extensive particle aggregation (hydrodynamic diameter > 1 μm), both of which reach their maximum above 5 mM CaCl_2_ (Figure 1C,D). For bR-nanodiscs at 2.5 mM Ca^2+^, only a low amount of lipid mixing is observed, however no increase in mean particle size is seen, suggesting that the lipid exchange interactions are transient in nature (Figure 1C,E). For higher Ca^2+^ concentrations, lipid mixing is further increased and is accompanied by the appearance of a second population of particles with hydrodynamic diameters of ∼30 nm. Notably, the bR-nanodiscs do not form very large aggregates, suggesting that they may be undergoing stacking^31,32^ as opposed to uncontrolled aggregation. This observation is confirmed by the reversible nature of this process; addition of a chelator results in reversion of the particle size to approximately that of the starting material (Figure S2). Mass photometry provided additional confirmatory results (Figure S3) where, after incubation with Ca^2+^, the number of lipids present in nanodiscs (estimated by mass) drops from 148 ± 58 to 117 ± 40 and then to 102 ± 36 after chelation of the metal. This shows that in the presence of calcium, nanodiscs are reshaped rather than aggregate. The higher masses measured in the samples and the large particle population seen in DLS can be attributed to the formation of nanodisc stacks. Finally, we observe that the bR-nanodiscs fuse more readily with liposomes, than with other bR-nanodiscs of the same “delivery” 2, requiring only 2.5 mM of Ca^2+^ (Figure 1C).

Taken together, these results suggest that contrary to what is seen for liposomes, bR-nanodiscs cross-interact transiently and are not prone to aggregate in an uncontrolled fashion upon addition of calcium, while also showing higher preference toward fusion with liposomes. This also suggests that the nanodiscs are a more stable carrier of membrane proteins compared to liposomes and cell-derived vesicles,^14^ being capable of avoiding excessive cross-fusion even at high Ca^2+^ concentrations, this in turn may have useful practical implications.

**Figure 2:**
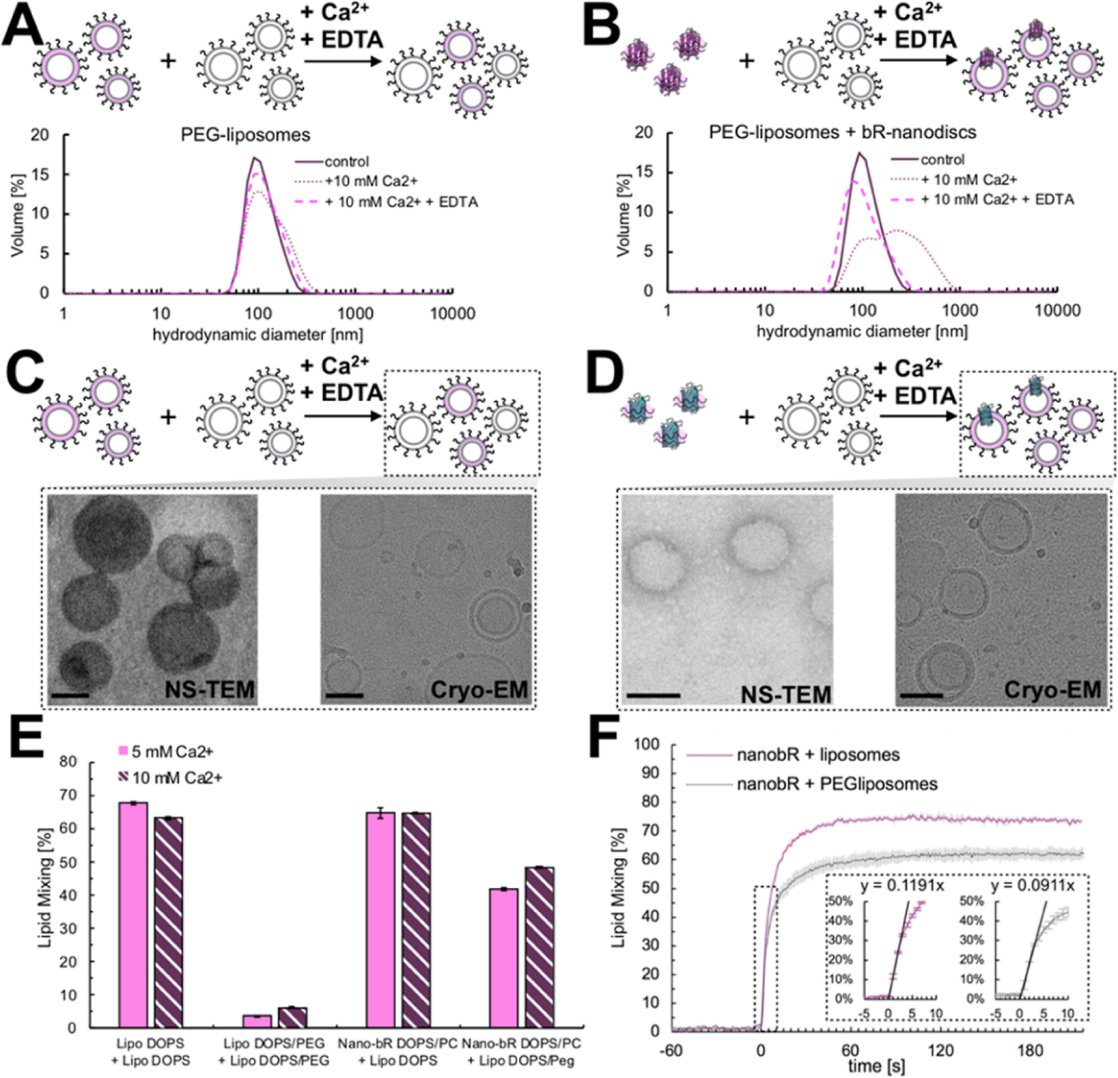
Size distribution changes upon the addition of Ca^2+^ and its subsequent removal using 50 mM EDTA for PEG-covered liposomes (A) and for PEG-covered liposomes mixed with bR-nanodiscs (B). Negative stain TEM (NS-TEM) images (left) and cryo-EM images (right) of postfusion vesicles in absence (C) and presence (D) of nanodiscs carrying OmpG. (E) Effect on lipid mixing of 2% 2K–PEG-PE in membranes of DOPS liposomes for SUV–SUV and bR–nanodisc–SUV fusion. (F) Kinetic measurements of bR–nanodisc–SUV fusion-induced at time 0 s with 10 mM CaCl_2_ for 2% 2K–PEG-PE coated (PEGliposomes) and pure DOPS liposomes. Insets show the initial response to calcium addition along with the initial fitted slopes. Labeled liposomes (DOPS/DOPC/Rhod-PE/NDB-PE; 96:2:2 or DOPS/DOPC/Rhod-PE/NDB-PE/PEG-2k-PE; 94:2:2:2) and bR-nanodiscs (DOPS/DOPC/Rhod-PE/NDB-PE; 83:13:2:2) were fused at a 1:9 ratio with either DOPS or DOPS/PEG-2k-PE (98:2) liposomes. Scale bars in the EM images are 100 nm.

To mitigate the second problem—liposome–liposome cross-aggregation—we decided to PEGylate the surface of the liposomes, as the addition of 2% of PEG-modified lipids has been shown to prevent peptide-driven liposome fusion.^33^ PEGylation of 2% of the lipids equates to ∼34% coverage of the liposome surface (Supporting Information), with the remaining ∼66% being readily available for fusion with nanodiscs. Indeed, our results show that, using PEG-2k-PE (1,2-dioleoyl-*sn*-glycero-3-phosphoethanolamine-*N*-[methoxy(polyethylene glycol)-2000]) at this coverage, Ca^2+^ driven fusion between liposomes was halted while bR-nanodisc-liposome fusion could proceed (Figure 2). The extent of lipid mixing for the bR-nanodiscs-liposome fusion slightly decreases upon introduction of PEG due to the lack of further dilution of the FRET pair (Figure 2E) arising from liposome cross-aggregation. However, the kinetics of fusion (Figure 2F) remain similar with initial slopes of ∼1/8 s (without PEGylation) and ∼1/11 s (with PEGylation), exceeding the reported rate of SNARE driven fusion by a factor of ∼360 for noncoated liposomes and ∼270 for PEGylated ones.^34^

DLS measurements show that the size of observed PEGylated liposomes is not significantly altered after undergoing Ca^2+^-driven fusion (with subsequent EDTA chelation) with bR-loaded MSP1E3D1 nanodiscs (Figure 2A,B). Additionally, NS-TEM, and cryo-EM imaging of the postfusion PEGylated liposomes with and without the addition of membrane protein-containing nanodiscs (here outer membrane protein G, OmpG, a bacterial porin) showed similar vesicles (Figure 2C,D), confirming that fusion with nanodiscs does not result in destabilization or significant changes in vesicle morphology.

**Figure 3:**
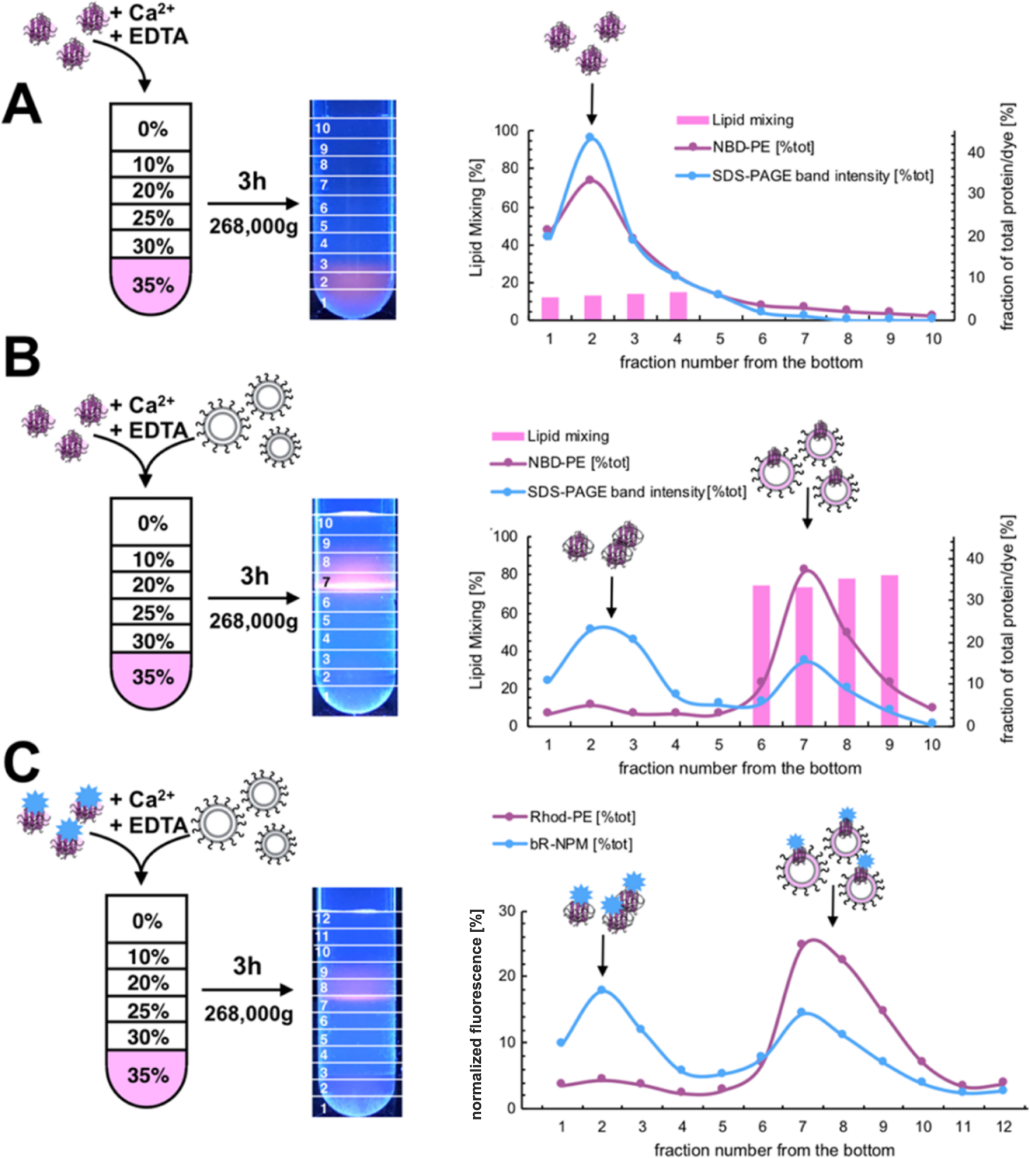
Ultracentrifugation analysis of postfusion products. Schemes of sample preparation are shown along with photographs showing the location of the fluorescent Rhod-PE signal after ultracentrifugation (approximate fraction positions are overlaid and numbered as collected). Comparison of ultracentrifugation of (A) DOPS/PC/Rhod-PE/NBD-PE (85:11:2:2) MSP1E3D1-bR nanodiscs and (B) MSP1E3D1-bR nanodiscs mixed with 100 nm DOPS/(18:1)-PEG-2k-PE (98:2) SUV liposomes undergoing fusion. Measured % of lipid mixing for corresponding fraction numbers (bars), normalized % of 530 nm fluorescence after detergent disruption found in the given fraction and distribution of total protein found in the fraction as established by densitometric analysis of SDS-PAGE gels (Figures S5 and S21) is shown. (C) Analysis of distribution of NPM labeled bR in fractions after ultracentrifugation of postfusion mixtures of MSP1E3D1-bR nanodiscs (DOPS/DOPC/Rhod-PE; 87:11:2) and SUV liposomes (DOPS/(18:1)-PEG-2k-PE). Distribution of 570 and 345 nm fluorescence measured showing the location of lipids and bR originally present in nanodiscs. Lines are added to the images to guide the eye.

To further study the liposomes prepared using nano-CRAFT we employed a typical ultracentrifugation in sucrose gradient which enables separation of the postfusion vesicles from nanodiscs and possible nanodisc-lipid aggregates (Figure 3) based on differences in their densities.

After ultracentrifugation, nanodiscs remain at the bottom of the tube as shown by the fluorescence of NBD-PE, while the liposomes, due to their hollow insides resulting in higher buoyancy, float up and are trapped near the 10/20% sucrose interface (Figure S4). Additionally, bR-nanodiscs after 30 min incubation with calcium and with subsequent fusion halted by addition of EGTA/EDTA, also do not change their position in the gradient (Figure 3A).

Conversely, when the incubation mixture included SUV liposomes, the majority of the signal from NBD-PE originally present in the nanodiscs moved to the fractions characteristic of liposomes (Figure 3B). Moreover, SDS-PAGE analysis of the protein content of collected fractions shows that ∼39% of total protein also moved to this fraction (Figure S5). This is accompanied by NDB-PE dequenching (lipid mixing), confirming that fusion is responsible for the observed transfer. Due to the MSP1E3D1 and bR having similar molecular weight, we could not discern if one or both proteins moved into the liposome fraction.

To specifically check for the transfer of membrane proteins, we fluorescently labeled the bR with NPM [*N*-(1-pyrenyl)maleimide] to track it directly and repeated the ultracentrifugation experiment. The results align well with the SDS-page data analysis and show that ∼52% of NPM signal is present in the liposomal fraction, as shown by colocalization with the signal from Rhod-PE (originally present in nanodiscs) transferred to liposomes (Figure 3C). The result of an independent preparation without Rhod-PE used for liposome positioning in the sucrose gradient similarly yielded ∼41% (Figure S6).

The discrepancy between the almost complete transfer of lipids and partial transfer of proteins can be explained by the events where postfusion, after the lipid exchange, some nanodiscs are detached from the liposomes and MSPs and bR constituting them are found in the higher sucrose “nanodiscs” fractions (Figure 3B,C). This behavior is consistent with dynamic nature of apolipoprotein AI (ApoAI; template for MSP) and its lipid shuttling action.^35,36^ For the postfusion fate of the MSPs found in the liposomal fraction there are two plausible pathways: one is that MSPs are retained on the surface of liposomes retaining the belt-like arrangement around the bR; second, is that the MSPs adopt a more open conformation releasing the membrane protein and lipid cargo in a fashion similar to that reported for ApoAI.^37^ We employed molecular dynamics (MD) simulations to investigate this question, using the coarse-grained Martini force field^38^ which is capable of catching realistic fusion events.^39,40^ We found that a possible fusion pathway for individual nanodiscs required removal of MSP for the lipid mixing to reflect experimental data (Supporting InformationFigures S7–S14). This points toward strong opening of MSPs being necessary for the observed lipid and membrane protein delivery.

Next, we tried to detect and quantify full fusion between MSP1E3D1-bR nanodiscs and PEGylated-liposomes. Full fusion events, where both leaflets of carrier nanodiscs would fuse with the target liposome, are the only events expected to result in the membrane protein being correctly inserted into the membrane. To this end we have employed a dithionite quenching assay (Figure S15), where the amount of lipids transferred to the inside leaflet of target membrane can be established upon addition of membrane impermeable sodium dithionite which converts NBD-PE to nonfluorescent ABD-PE (7-amino-2,1,3-benzoxadiazol-4-yl PE analog).^41^ In such experiments the fluorescent signal of NBD-PE measured after the addition of dithionite is proportional to the fraction of lipids present in the inner leaflet of the liposome and referred to as “full fusion.” The extent of full fusion (Figure S16) was found to be ∼21% with ∼63% total fusion for the sample as whole (i.e., prior to separation by ultracentrifugation). This number is in excellent agreement with the data acquired for fluorescently labeled bR: the ∼21% of fluorescence signal measured after quenching suggests twice that amount (∼42%) of nanodiscs in the sample have undergone complete fusion and delivery the membrane protein.

To gauge how limiting the area of fusogenic lipids in nano-CRAFT system would influence the various transfer efficiencies, we used the same dithionite quenching and ultracentrifugation setup to test the fusion of smaller, MSP1D1-based nanodiscs (*d*_h_ ≅ 9.7 nm)^15^ containing a single bR and MSP1E3D1 nanodiscs having trimeric bR (Tables S3 and S5 and Figures S17–S22). In those the proteins are expected to occupy over 14 and 21% of the membrane, respectively.

The extent of fusion and lipid transfer in MSP1D1-based nanodiscs is similar to their larger (MSP1E3D1) counterpart, with their ability to transfer proteins being diminished. The presence of bR trimers in MSP1E3D1 results in a twofold decrease in all measured parameters of fusion compared to monomeric bR. However, for all studied systems, the ratio between full fusion and total fusion is comparable (Table S3) and the lipid mixing measured in postfusion products separated using ultracentrifugation is similar (∼74 ± 4%; Table S4). This implies that the ratio between the area of fusable lipids present in nanodiscs and the area occupied by the membrane protein (Figure S22) dictates the overall fusion efficiency, by limiting the number of fusion events, rather than extent of fusion for individual particles. Taken together, this suggests that, when using nano-CRAFT, the choice of nanodisc size, as compared to the target membrane protein to be delivered, is crucial to ensure maximal transfer efficiency.

**Figure 4:**
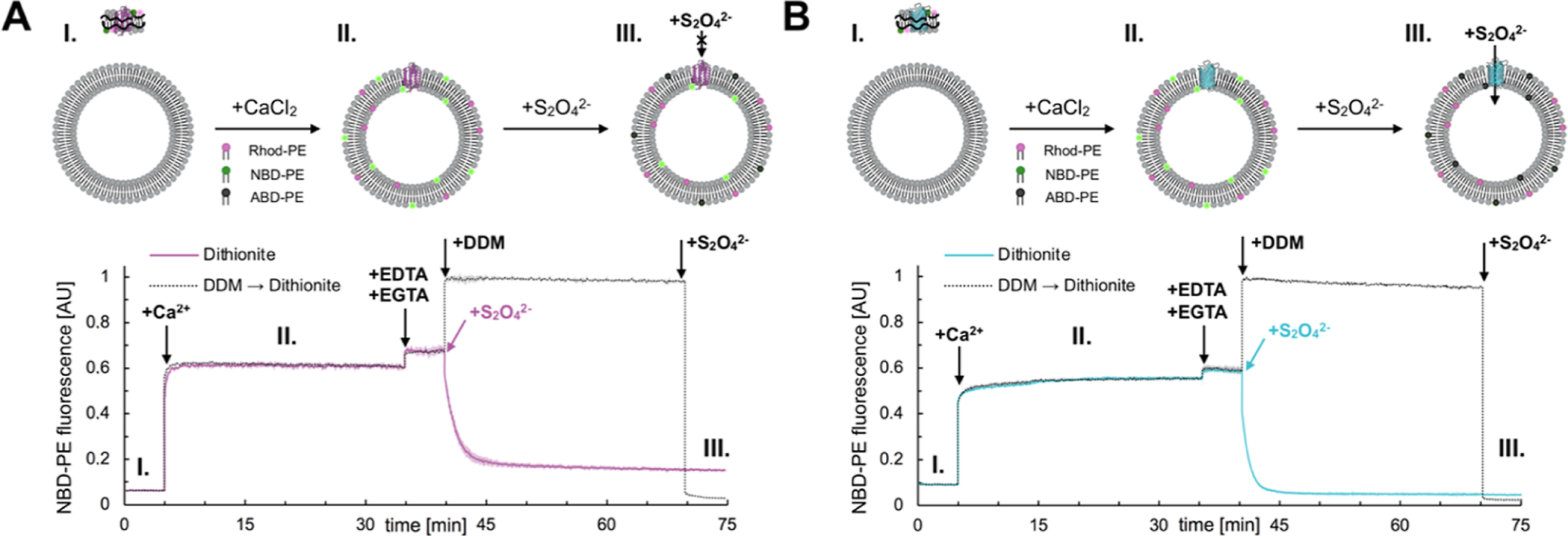
Comparison of membrane permeability to dithionite for liposomes after fusion with bR–nanodiscs and OmpG–nanodiscs. Time course measurements showing the extent of normalized NBD-PE fluorescence prior the initiation of fusion (I), postfusion (II) and after dithionite addition (III) are shown for bR-nanodiscs (A) or OmpG containing nanodiscs (B) (DOPS/DOPC/Rhod-PE/NBD-PE; 85:11:2:2) fusion with DOPS/PEG-2k-PE (98:2) liposomes. The experimental curves (solid lines) and control curves reporting the maximal (detergent disruption) and minimal (detergent disruption followed by dithionite quenching) fluorescence possible (dotted lines) are shown. Postfusion (II) the NBD-PE fluorescence is comparable for OmpG and bR nanodiscs reporting a similar amount of lipid mixing. After addition of dithionite (III) in the case of bR the fluorescence signal is not completely quenched due to presence of lipids transported into the inside leaflet of liposomes, whereas for OmpG those lipids are quenched by dithionite penetrating the bilayer via the protein’s pore (schemes on the top). Here fusion was stopped using 5 equiv of EDTA and 2 equiv of EGTA.

To confirm whether the delivered proteins are indeed correctly inserted in a membrane spanning fashion, we employed nanodiscs carrying a bacterial porin, OmpG, which, after fusion, would make the membrane permeable to dithionite by virtue of introducing pores into the membrane, resulting in enhanced quenching of NDB-PE fluorescence. Indeed, while measured lipid mixing reported by the NDB-PE dequenching upon addition of calcium (∼55%; stage II in Figure 4B) was comparable to nanodiscs containing bR (∼61%; stage II in Figure 4A) as expected by the area occupied by the OmpG (Figure S22), the dithionite almost completely quenched the fluorescence of NBD-PE, showing that the membranes of SUVs became dithionite permeable (Figure 4, stage III) and confirming trans-membrane insertion of delivered proteins.

Having shown that our nano-CRAFT system can be utilized for delivery of membrane proteins into SUVs we next tested if it could be used to fuse nanodiscs with cell-sized lipid vesicles, that is, GUVs. The lipid composition of “target” GUVs had to be changed, as electroformed GUVs formed from pure DOPS showed poor yields. The final composition used was 75% DOPS, 23.5% DOPC, and 1.5% Cy5-PE [1,2-dioleoyl-*sn*-glycero-3-phosphoethanolamine-*N*-(Cyanine 5); used for GUV positioning during imaging]. Initially we tested fusion between “delivery” MSP1E3D1 nanodiscs (Table S1) carrying OmpG using our standard lipid mixing setup. Postfusion dequenching of NBP-PE in the receiving GUV membrane (Figure S23) was observed showing that nanodisc–GUV fusion was possible. Crucially, the same dequenching was not observed when nonfusable DOPC-based GUVs were used.

To further investigate if nano-CRAFT can be used to enrich preformed GUV membranes with OmpG, we compared the effects on GUV membrane permeability of NBD-modified glucose (2-NBDG) after fusion with MSP1E3D1 nanodiscs with and without OmpG. For these experiments, the nanodiscs included 0.5% Rhod-PE as the only dye as the green channel was used for monitoring the 2-NBDG (2-(*N*-(7-nitrobenz-2-oxa-1,3-diazol-4-yl)amino)-2-deoxyglucose) loaded inside the GUV membranes (Table S1).

**Figure 5:**
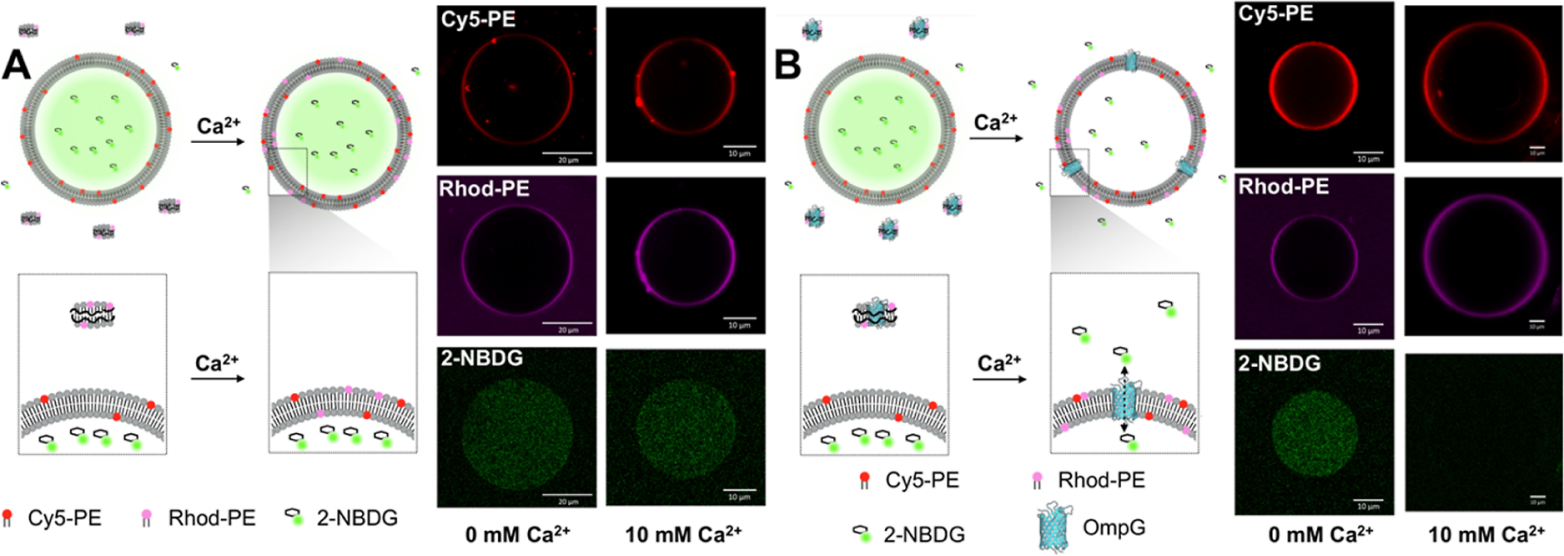
Confocal imaging of fusion of nanodiscs (A) and nanodiscs bearing an OmpG (B) with GUVs. After the addition of 10 mM Ca^2+^ the Rhod-PE signal is completely colocalized with the Cy5-PE signal used for GUV positioning. Additionally, the signal for 2-NBDG loaded inside GUVs disappears from the inside of GUVs after fusion with OmpG-nanodiscs, showing correct membrane protein insertion. The nanodisc lipid composition was to DOPS/DOPC/Rhod-PE (75:24.5:0.5) with GUVs being composed of DOPS/DOPC/Cy5-PE (75:23.5:1.5).

Prior to the addition of 10 mM Ca^2+^ in both the control “empty” nanodiscs and the OmpG nanodiscs, some accumulation of Rhod-PE signal could be seen on the periphery of the GUV as well as a steady background surrounding the GUV, indicating the presence of free-floating nanodiscs in the solution (Figures 5 and S24). After the fusion, in both setups, the Rhod-PE signal is almost completely moved to the periphery of GUVs, suggesting lipid transport to the membranes. In the case of OmpG-containing nanodiscs (Figure 5B), this is accompanied by the loss of the 2-NBDG contents of the GUVs, as opposed to the empty nanodisc, where the 2-NBDG remains (Figure 5A). This shows that the OmpG was introduced into GUV membranes in a transmembrane spanning fashion (Figure 5B), rendering them permeable to 2-NBDG. Additionally, the retention of 2-NBDG in GUVs after fusion with empty nanodiscs shows that the fusion process itself happens without substantial content release. This may be useful for synthetic biology applications, where the retention of material loaded inside GUVs may be crucial. Content release was independently tested for membrane protein-loaded nanodiscs using a buoyancy assay (Figure S25) and was shown to be no higher than 35% for SUVs.

## Conclusion

In this work, we have developed a novel one-pot method, named nano-CRAFT, for the delivery of membrane proteins to preformed lipid vesicles using nanodisc-liposome calcium-driven fusion. This method proved suitable for modification of both small (100 nm) and giant size (20–100 μm) liposomes and is able to deliver membrane proteins in a proper membrane-spanning fashion while avoiding vesicle aggregation by utilization of PEGylation of the liposome surface. Given that the sizes of SUVs and GUVs fall within the range desirable for artificial cells and subcellular compartments, respectively, the method may prove useful for engineering both the outer membranes of artificial cells, as wells as the membranes of subcellular artificial organelles. Additionally, nano-CRAFT allows the membrane proteins used for bottom-up construction of artificial cells to be stored embedded in nanodiscs, which are known to have excellent storage stability.^42^

Crucially, employing nanodiscs makes the delivery protocol independent of detergent, which in turn can make preparation of more complex artificial cells easier, as the proteins of interest are in only contact with detergents which are suitable for them. Moreover, the proposed fusion system should be applicable to construction and assembly approaches that use both sequential addition and simultaneous introduction of many membrane proteins. The latter feature is possible due to the limited cross-interactions and strong preference toward fusion with liposomes shown by nanodiscs. Additionally, the rapid kinetics of the fusion, only requiring a transient (∼4 and ∼30 s for 50 and 90% completion respectively, Figure 2F) exposure to Ca^2^, can be advantageous for systems where prolonged exposure to Ca^2+^ would be detrimental and can be used in single-pot reactions but also readily adapted in rapid buffer exchange systems in microfluidics setups.^43^ Moreover, the rate of this transfer considerably outperforms cell penetrating peptides, SNARE- and DNA-based alternatives.^21,22,34,44,45^ Finally, as opposed to delivery approaches^12,13,46,47^ where the membrane proteins have to be incorporated into unnatural positively charged membranes, in our setup the proteins are incorporated into negatively charged bilayers which are more suited for handling both prokaryotic and eukaryotic proteins alike as they more closely reflect the charge of naturally occurring membranes.^48−50^ The addition of lipids essential for target membrane protein can also be achieved, as the fusion does not require nanodiscs to be composed solely of PS.

While nano-CRAFT in itself allows for easy and efficient fusion with target membrane, we believe that our method can be extended even further beyond the presented applications via facile modification of MSP proteins made possible by the presence of numerous lysine residues, free N- and C-termini and the absence of cysteine.^51−53^ These provide attachment points that have the potential to be used for the coupling of membrane interacting moieties such that nanodisc orientation could be controlled during the fusion process. Further, by using the well-established high compatibility of nanodiscs with DNA nanoscience^54−56^ such attachment points may also be integrated with DNA structures to constrain individual embedded membrane proteins such that the orientationally controlled insertion of protein into target membranes is achieved. In this case, the utilization of nanodiscs ability to carry singular membrane proteins is crucial as this feature is not found in vesicle-based fusion delivery methods. Work to achieve this goal is ongoing.

We also believe that the nano-CRAFT system can be built upon so that eventually, different compartments might be modified with different membrane proteins in a precisely controlled fashion, bringing the goal preparation of intricate artificial cells a step closer to reality.

## Methods

DOPS-Na (1,2-dioleoyl-*sn*-glycero-3-phospho-l-serine, sodium salt, COATSOME MS-8181LS) was purchased from NOF Japan. The remainder of the phospholipids and other compounds were purchased from Merck KGaA.

### Small Unilamellar Vesicle Preparation Via Extrusion

Liposomes were prepared using a standard mini-extruder preparation method. Extrusion consists of passing the liposomes through a filter of known pore size under pressure where the resulting liposome solution has a homogeneous size due to selectivity imposed by the pore. Briefly, a lipid film containing 1 μmol of chosen lipids was prepared by drying chloroform stock solutions of lipids in a glass vial using a gentle stream of argon and subsequently left under high vacuum for at least 4 h to ensure removal of residual organic solvent. Following this, the lipid film was hydrated by the addition of 20 mM Tris 100 mM NaCl and 0.5 mM EDTA at pH 7.4 and carefully vortexed until no lipid residue was found on the walls of the glass vial. The resulting opaque suspension of multi-lamellar vesicles next underwent five freeze–thaw cycles using liquid nitrogen. For pyranine encapsulation experiments, this was extended to 10 cycles. Next, liposomes were extruded using an Avanti Mini-Extruder equipped with Whatman Nuclepore Track-Etched Membranes, 0.1 μm, by passing the liposome suspension through the extruder 13 times. The size and homogeneity of the resulting particles were checked for every preparation using dynamic light scattering. For pyranine encapsulation experiments, the liposomes were purified from excess dye using HiTrap desalting columns (GE Healthcare). Liposomes were typically prepared and used on the same day. The full list of lipid composition used for liposomes preparations is presented in Table S1.

### Giant Unilamellar Vesicle Preparation Via Electroformation

GUVs are typically in the 1–200 μm size range and are widely used as model cell membranes. In electroformation, during lipid film hydration, an alternating electric field is applied to the sample facilitating the GUV formation process. A 10-μL drop of a 2-mM chloroform stock solution of a chosen lipid mix was spread on conductive ITO-coated glass slides (resistance 50 Ω, Nanion Technologies GmbH) and left to dry for at least 15 min at room temperature. Then the ITO-coated slide containing the lipid film was assembled with a second slide using a rubber O-ring spacer to form an approximately 300 μL chamber that was filled with a solution containing 360 mM sucrose, 1 mM HEPES and with/without 2 μM 2-NBDG (pH 7.4, osmolarity ∼ 360 mOsmol/L). Electroformation was carried out by using a Vesicle Prep Pro device (Nanion Technologies GmbH).

The electroformation protocol for the DOPS-based lipid composition no. 5 (Table S1) had a rise time of 60 min at a frequency of 20 Hz and a voltage ramp from 0 to 3.2 V (peak-to-peak voltage, sinusoidal wave shape); a main (formation) time of 90 min at a frequency of 20 Hz and a fixed voltage of 3.2 V; and a fall time of 15 min with a frequency ramp from 20 to 4 Hz at a fixed voltage of 3.2 V. All steps were performed at 37 °C.

The electroformation of DOPC-based GUVs (lipid composition 5Ø, Table S1) was carried out at a frequency of 10 Hz and at 3 V (peak-to-peak voltage, sinusoidal wave shape) with a rise time of 5 min, main (formation) time of 60 min, and a fall time of 10 min, with all steps performed at 37 °C.

### Expression and Purification of MSPs

Plasmids for expression of MSP1D1 and MSP1E3D1 were a gift from S. Sligar (Addgene plasmid nos 20061 and 20066) and were expressed and purified according to the previously published protocol.^15^

### Expression and Purification of bR

A modified version of the plasmid for overexpression of Mistic-bacterioopsin fusion protein was designed based on a previously published protocol.^57^ The construct consists of a *Bam*HI restriction site and Mistic protein (Uniprot accession number: Q5BU39), thrombin cleavage site (LVPRGS), QDVL, ybbR-tag sequence (DSLEFIASKLA), TEV cleavage site (ENLYFQS), C138 (used for fluorescent labeling), bR (Uniprot accession number: P02945, starting at S13), S386, D387, and the XhoI restriction site. Mistic is a protein fragment known to increase expression of membrane proteins expressed in *Escherichia coli*. The DNA sequence for this was synthesized and cloned into pET-28a(+) using cloning sites NcoI/Xhol by Genscript Biotech Corp providing the protein with a 6× His C-terminal tag for further purification. The ybbR-tag sequence and TEV cleavage site were not utilized in this study. An additional variant was prepared where C138 was removed by site-directed mutagenesis polymerase chain reaction using the following primer strands: forward–cctgtactttcagagccaggcgcaaatcaccg and reverse–cggtgatttgcgcctggctctgaaagtacagg. This cysteine-less variant was used for all experiments excluding ones where bR was labeled with *N*-(1-pyrenyl)maleimide. The proteins were purified and renaturated into bR using the protocol reported by Nekrasova et al.^57^ with slight alterations. The prepared protein stock was stored in 100 mM NaOAc, pH 4.5, 0.1 NaCl, and 0.2% DDM.

### Expression and Purification of OmpG

The gene encoding the mature OmpG was subcloned into pTAMAHISTEV.^58^ OmpG was overexpressed in *E. coli* BL21(DE3) Omp8 cells (Δlamb ompF::Tn5 ΔompA ΔompC) in LB medium. Outer membranes were prepared as before.^59^ In brief, OmpG was extracted in 1% LDAO and purified by Ni-NTA chromatography. The detergent was exchanged to 1% OG via size exclusion chromatography (20 mM Tris–HCl, 150 mM NaCl and 1.0% OG). Protein purity was assessed by SDS-PAGE.

### bR Labeling with *N*-(1-Pyrenyl)maleimide

bR(C138) was reduced by mixing the stock solution 1:1 with 50 mM NaP, 250 mM NaCl, 4 mM TCEP, 2 mM EDTA pH 8 buffer followed by 1 h incubation at room temperature. Excess of TCEP was removed, and the sample was moved to 50 mM NaP, 250 mM NaCl, 0.5 mM EDTA, and 0.1% DDM by at least four rounds of ultrafiltration using an Amicon Ultra-0.5 Centrifugal Filter Unit. Following this, 800 mL of 11.5 μM bR(C138) was mixed with 200 mL of 10 mM *N*-(1-pyrenyl)maleimide (Sigma-Aldrich) and was dissolved in DMSO. The reaction was left overnight at room temperature. Next the precipitated label was removed by centrifugation, and supernatant was collected. Excess DMSO was removed by two rounds of ultrafiltration and by passing the samples through a HiTrap (GE healthcare) desalting column in 50 mM NaOAc; 100 mM NaCl with 0.1% DDM 0.1% pH 4.5. The prepared protein conjugate was used for nanodisc preparation, as described below. Note that during the nanodisc preparation any unreacted *N*-(1-pyrenyl)maleimide (Sigma-Aldrich) remaining in the sample is removed during the Amberlite XAD-2 (Supelco) treatment, His-Tag, and Size Exclusion Chromatography purification steps.

### Nanodisc Preparation

Nanodiscs were assembled using a previously published protocol.^60^ Briefly, the lipid film containing a 6-μmol mix of chosen lipids was prepared as described in the liposome/vesicle preparation section. The film was then hydrated using a 20 mM Tris, 100 mM NaCl, 0.5 mM EDTA, pH 7.4 buffered solution of 100 mM sodium cholate and was thoroughly vortexed until the solution became clear. Next, membrane protein of choice and 20 mM Tris, 100 mM NaCl, 0.5 mM EDTA pH 7.4 (up to desired volume) was added. The ratios used for the preparation of different variants of nanodiscs were established experimentally based on the expected nanodisc size and area per lipid of DOPS and were as follows:

Lipid only MSP1E3D1 nanodiscs—1:100 MSP1E3D1:lipids.

Monomeric bR MSP1E3D1 nanodiscs—1:5:500 bR/MSP1E3D1/lipids.

Trimeric bR MSP1E3D1 nanodiscs—3:2:108 bR/MSP1E3D1/lipids.

Monomeric bR MSP1D1 nanodiscs—1:10:540 bR/MSP1D1/lipids.

Monomeric OmpG nanodiscs—1:2:100 OmpG/MSP1E3D1/lipids.

Following this, the sample was incubated for 30 min at 28 °C. Next MSP1E3D1 or MSP1E3D1 was added followed by additional 5 min of incubation at 28 °C. The final volume of preparation was typically 1 mL with 6 mM lipid and the final sodium cholate concentration being 24–50 mM. Next, the nanodisc assembly was initiated by the addition of Amberlite XAD-2 (Supelco) at 0.5 mg/mL and incubation with shaking at 28 °C for 3 h. Following this, the sample was moved to a fresh bead batch (0.5 mg/mL), and the incubation continued for an additional 1 h. For bR- and OmpG-loaded nanodiscs, the empty nanodiscs were removed by using His-tag affinity chromatography. Next, the samples were further purified from free MSP and possible aggregates on a Superdex 200 Increase 10/300 GL (Cytiva) with a running buffer of 20 mM Tris, 100 mM NaCl, 0.5 mM EDTA, pH 7.4. The quality of preparations was assessed using SEC with peak fractions were further tested using DLS. Nanodiscs were used not later than 7 days after preparation. Full list of lipid composition used for nanodiscs preparations is presented in Table S1.

### Fluorescence Measurements

Fluorescence measurements were conducted using a Tecan infinite 200Pro plate reader and Corning 96 NBS black polystyrene well plates. For lipid mixing steady-state experiments, the excitation was set to 450 nm with 10 nm bandwidth and emission measured in 2 or 5 nm increments starting from 480 to 720 nm. For analysis of postultracentrifugation fractions for Rhod-PE, the excitation was set to 540 nm with 10 nm bandwidth and the emission was measured in 2 nm increments starting from 570 to 720 nm while for NPM-labeled bR the excitation was set to 325 nm with 10 nm bandwidth and emission measured in 2 nm increments starting from 355 to 549 nm.

For positioning of pyranine in postultracentrifugation samples, the absorbance of the samples was measured from 350 to 650 nm in 3 nm increments using a Tecan infinite 200Pro plate reader and Nunc EdgeTM 96-well, non-treated, flat-bottom microplates.

Time course measurements pertaining to full fusion/dithionite protection assays were performed using a Tecan infinite 200Pro plate reader and Corning 96 NBS black polystyrene well plates. The excitation was set to 450 nm with 9-nm bandwidth and the emission wavelength was set to 530 nm with 20-nm bandwidth. The interval time between measurements was set to minimal.

The time course measurements measuring the initial rates of fusion were conducted on a RF-6000 fluorescence spectrofluorometer (Shimadzu) using a 1.5 mm × 1.5 mm light patch quartz glass cuvette. The excitation and emission wavelengths were set to 450 and 530 nm, respectively, both with bandwidths of 3 nm, cycle time of 1 s, and accumulation time set to 500 ms. All the experiments were conducted at 25 °C.

Fusion experiments were carried out at a 1:9 molar ratio of lipids present in labeled and unlabeled particles, respectively (unless stated otherwise), with the final lipid concentration of labeled particles being 62.5 μM. Fusion was initiated by addition of 10 mM calcium (unless stated otherwise) followed by 30 min incubation with subsequent chelation (if stated) using EDTA and/or EGTA. Lipid mixing was measured by comparing the 530 nm fluorescence of postfusion sample to its fluorescence after addition of 0.5% DDM. Full fusion was measured based on a dithionite quenching assay where 530 nm fluorescence was measured 12 min after the addition of 100 mM of sodium dithionite freshly dissolved in 100 mM Tris (pH 10) and was compared to a duplicate sample treated with 0.5% DDM. The exact formulas used to calculate extent of FRET, lipid mixing, and full fusion are presented in Supporting Information.

### Ultracentrifugation

Ultracentrifugation allows samples to be separated by mass and density upon application of a centrifugal force. Samples for ultracentrifugation experiments were prepared at a ratio of 1:9 labeled/unlabeled with the final lipid concentration of labeled particles being 267 μM. The samples were supplemented with 10 mM CaCl_2_ and incubated for 30 min. Calcium was subsequently chelated using 5 equiv of EDTA and 2 equiv of EGTA. For loading in the ultracentrifugation tube, the samples were prepared by mixing 1:1 with 70% sucrose solution using a 20 mM Tris 100 mM NaCl 0.5 mM EDTA pH 7.4 buffered solution and placing 400 μL of the resulting 35% gradient layer in the bottom of an open-top thickwall polycarbonate tube (3.5 mL, 13 × 51 mm; Beckman Coulter). Following this, 400 μL layers of buffer 30, 25, 20, 15, 10% sucrose solutions were carefully layered in 200 μL increments to minimize mixing. The final layer consisted of ∼400 μL of buffer with volume adjusted as needed to balance the centrifuge tubes. The samples were then spun for 3 h at 268,000*g* in a precooled (4 °C) Optima MAX-XP (Beckman Coulter) centrifuge. After the centrifugation, fractions were collected from the bottom of the tube using a long needle connected to an ÄKTA Start (GE Healthcare) chromatographic system.

### Dynamic Light Scattering

Dynamic light scattering measurements were conducted at 25 °C using a Zetasizer NANO ZSP (Malvern) in disposable UV microcuvettes using the same conditions as for fusion and full fusion measurements.

### Mass Photometry

Mass photometry is a relatively new technique that uses scattering of light to measure mass of molecules in solution. Mass photometry data were collected using a Refeyn OneMP instrument (Refeyn). The instrument was calibrated using a native marker protein standard mixture (NativeMark Unstained Protein Standard, Thermo Scientific), containing proteins in the range from 20 to 1200 kDa. Masses 66, 146, 480, and 1048 kDa were used to generate a standard calibration curve. Prior to the measurements, the borosilicate coverslips were extensively cleaned with Milli-Q water and isopropanol. One microliter of sample prepared using same condition as for fluorescence measurements was applied to 9 μL buffer on a coverslip resulting in a final particle concentration of 10 nM. Movies were acquired by using AcquireMP (Refeyn) software for 60 s with a frame rate of 1000 Hz and frame binning of 10 (effective frame rate 100 Hz). All data were processed with DiscoverMP (Refeyn) software. Threshold 1 and threshold 2 parameters were 1.50 and 0.25, respectively. Frame binning for the ratiometric frame calculation was 5. Masses were estimated by fitting a Gaussian distribution into mass histograms and taking the value as the median of the distribution. The number of lipids in nanodiscs was estimated by subtracting the masses of bR (*m*_bR_ 30.7 kDa) and two MSP1E3D1 (*m*_MSP1E3D1_ = 30 kDa) from the measured median particle mass and diving the acquired value by average mass of lipid in the nanodisc (0.804 kDa = 75% *m*_DOPS_ + 25% *m*_DOPC_).

### Confocal Microscopy

For visualization, GUVs were collected after the electroformation procedure and transferred to an imaging chamber containing a glass slide previously functionalized with 1 mg/mL bovine serum albumin. The ionic composition of the external solutions used for these experiments was 100 mM NaCl, 10 mM CaCl_2_, 20 mM Tris-HCl and 85 mM glucose (pH 7.4, osmolarity ∼ 360 mOsmol/L). For control experiments, CaCl_2_ was replaced with an equimolar osmotic concentration of glucose. Confocal Microscopy was performed with a Zeiss LSM 880 confocal microscope using a Plan-Apochromat 20× objective. The excitation/emission profile for each of the fluorophores used was as follows: 2-NBDG or NBD-PE was excited with a 458 nm Argon laser and emission collected at 525 nm, Rhod-PE was excited with a 561 nm diode-pumped solid-state laser and emission collected at 583 nm, and CY5 was excited with a 633 nm HeNe laser and emission collected at 664 nm. Image processing was performed using ZEN 3.3 (blue edition) and ImageJ.

### NS-TEM and Cryo-EM

The samples for both negative stain (NS) TEM and cryo-EM were prepared at ∼20 nM liposome particle concentration. The samples were supplemented with 10 mM CaCl_2_ and incubated for 30 min and subsequently chelated using 5 equiv of EDTA and 2 equiv of EGTA.

For NS-TEM, the Formvar/carbon-coated grids, 400 mesh copper grids (EM Resolutions) were subjected to glow discharge prior to sample application. Next, 5 μL of sample was applied to the grid and incubated for 2 min and blotted using filter paper. Subsequently, 5 μL of uranyl acetate (3%) was applied to the grid and immediately blotted followed by a second 5 μL application, 1 min incubation, and final blotting of the grid. The grids prepared in this way were visualized using a JEOL JEM-1230 80 kV instrument.

Samples for cryo-EM were prepared by application of 3 μL of postfusion mixture to a glow discharged ultrathin carbon on Lacey carbon (400 mesh) supported copper grids which were plunge-frozen in liquid ethane by FEI Vitrobot. The parameters used were: blot force 8, blot wait time of 4 and 30 s, respectively. The Vitrobot chamber temperature was set to 10 °C and humidity to 100%. The grids were visualized using Glacios cryo-EM (Thermo Scientific) equipped with a Falcon 4 direct electron detector.

## Supporting Information Available

Lipid compositions and their combination used for fusion experiments, equations used for determining FRET, lipid mixing, and full fusion, calculations for establishing the PEG coating on the surface of liposomes, calibration curve for lipid mixing assays alone, DLS and mass photometry data, additional, ultracentrifugation experiments along with analyses of separated products: protein transfer with fluorescence and SDS-PAGE (including densitometric traces), lipid mixing and full fusion fluorescence curves, visualization of different nanodiscs studied, tables summarizing the fusion efficiency results for various nanodiscs, additional confocal microscopy experiments, dye leakage from liposome during fusion using encapsulated pyranine, supplementary MD methods, results, and discussion (PDF)

## Abbreviations

SUV: small unilamellar vesicle
GUV: giant unilamellar vesicle
MSP: membrane scaffold protein
bR: bacteriorhodopsin
OmpG: outer membrane protein G
ApoAI: apolipoprotein AI
DOPS: 1,2-dioleoyl-*sn*-glycero-3-phospho-l-serine
DOPC: 1,2-dioleoyl-*sn*-glycero-3-phosphocholine
PEG-2k-PE: 1,2-dioleoyl-*sn*-glycero-3-phosphoethanolamine-*N*-[methoxy(polyethylene glycol)-2000]
NBD-PE: 1,2-dioleoyl-*sn*-glycero-3-phosphoethanolamine-*N*-(7-nitro-2–1,3-benzoxadiazol-4-yl)
Rhod-PE: 1,2-dioleoyl-*sn*-glycero-3-phosphoethanolamine-*N*-(lissamine rhodamine B sulfonyl)
Cy5-PE: 1,2-dioleoyl-*sn*-glycero-3-phosphoethanolamine-*N*-(cyanine 5)
2-NBDG: 2-(*N*-(7-nitrobenz-2-oxa-1,3-diazol-4-yl)amino)-2-deoxyglucose
FRET: Förster resonance energy transfer
DLS: dynamic light scattering
NS-TEM: negative stain transmission electron microscopy
cryo-EM: cryogenic electron microscopy
NPM: *N*-(1-pyrenyl)maleimide
MD: molecular dynamics
