# Supplementary material for: CRAFTing Delivery of Membrane Proteins into Protocells using Nanodiscs
Source: ACS Appl Mater Interfaces. 2023 Nov 28;15(49):56689–701. doi: 10.1021/acsami.3c11894 (PMC10726305; doi:10.1021/acsami.3c11894)
Supplement: Supplementary file 1 — am3c11894_si_001.pdf [file am3c11894_si_001.pdf]

CRAFTing Delivery of Membrane Proteins into Protocells Using Nanodiscs

Piotr Stępień[1], Sylwia Świątek[1], Manuel Yamil Yusef Robles[1], Joanna Markiewicz-Mizera[1], Dhanasekaran Balakrishnan[1], Satomi Inaba-Inoue[2,3†], Alex H. de Vries[4], Konstantinos Beis[2,3], Siewert J. Marrink[4] and Jonathan G. Heddle[1,†]

1. Malopolska Centre of Biotechnology, Jagiellonian University, Krakow, 30-387, Poland.
2. Department of Life Sciences, Imperial College London, Exhibition Road, South Kensington, London SW7 2AZ, UK.
3. Rutherford Appleton Laboratory, Research Complex at Harwell, Didcot, Oxfordshire OX11 0FA, UK.
4. Groningen Biomolecular Sciences and Biotechnology Institute, University of Groningen, 9747 AG, Groningen, The Netherlands

[†]Present address: School of Biological and Biomedical Sciences, Durham University, Durham DH1 3LE, UK

Table of Contents

Fluorescence based fusion experiments ***3***

FRET efficiency 3

Lipid mixing assay 3

Full fusion/dithionite protection mixing assay 3

Additional comment on fluorescence measurement 4

Estimation of PEG coating on the surface of liposomes ***4***

Coarse Grain (CG) simulations of an individual fusion event ***5***

CG Methods 6

CG Results: Radial stalk expansion leads to hemifused state with inverted micelle 7

CG Results: Unwrapping MSP allows fusion to proceed 8

CG Results: Rupturing the inverted micelle to deliver bR 8

CG Discussion 8

Supplementary Data ***11***

Table S1 11

Table S2 11

Figure S1 12

Figure S2 13
Figure S3 14
Figure S4 15
Figure S5 16
Figure S6 16
Figure S7 17
Figure S8 18
Figure S9 18
Figure S10 19
Figure S11 19
Figure S12 20
Figure S13 20
Figure S14 21
Figure S15 22
Figure S16 23
Figure S17 24
Figure S18 25
Figure S19 26
Figure S20 27
Figure S21 28
Figure S22 29
Table S3 29
Table S4 29
Table S5 29
Figure S23 30
Figure S24 30
Figure S25 31
References *32*

Fluorescence based fusion experiments

FRET efficiency

For the prepared calibration curve (Figure S1) the FRET efficiencies (E) were calculated was follows[1]:

$$E = 1 - \frac{F}{C * F_{max}}$$

where the F is the 530 nm (NBD-PE) fluorescence of the sample in presence of given concentration of the acceptor (Rhod-PE) and F_{max} is the 530 nm fluorescence of the sample after disruption with detergent, which approximates infinite dilution of the lipids and thus absence of the acceptor. C is the experimental correction factor described in the next section.

For the calibration curve the lipid mixing corresponding to the FRET efficiencies was calculated as follows:

$$lipid\ mixing = \frac{F}{C * F_{max}} \cdot 100\%$$

Which is the simplified form of the equation used in fusion experiments described below.

Lipid mixing assay

The extent of fusion efficiency was calculated by measuring the dequenching of the 530 nm fluorescence of NBD-PE upon spatial separation from Rhod-PE present in the labelled nanodisc/liposome population (Figure 1A). After measuring the initial fluorescence, F_0, the fusion was initiated by addition of 10 mM $CaCl_2$ followed by a 30 minute incubation unless stated otherwise. After this the 530 nm fluorescence of sample was measured yielding F. Following this measurement, the samples were destroyed by addition of DDM. This approximated the infinite dilution of the lipids providing the reference value of 100% fusion F_{max}. As DDM can slightly alter the fluorescence of NBD-PE its effect on the quantum yield was corrected by measuring the fluorescence of DOPS liposomes containing 2% of NBD-PE (lipid composition 0, Table S1) before and after the detergent treatment.

The lipid mixing was calculated as follows:

$$lipid\ mixing = \frac{F - F_0}{C * F_{max} - F_0} \cdot 100\%$$

With C being the correction calculated for DDM for control sample for each set of experiments.

Full fusion/dithionite protection mixing assay

To measure the degree of full fusion, a dithionite quenching protocol was employed. Briefly, all samples were measured in duplicate. After measuring the initial 530 nm fluorescence intensity (F_{init}) 10 mM of sodium dithionite was added to one sample with 0.5% DDM being added to the second. Sodium dithionite as a 100 mM sodium dithionite stock solution prepared using 100 mM Tris pH 10 buffer was always freshly prepared prior to addition. The 530 nm fluorescence measured after 12 minutes provides values describing amount of lipid transfer into the interior of liposomes, F_{full} and F_{max} for the dithionite quenching and detergent disruption respectively. After this, freshly diluted sodium dithionite was added to the detergent disrupted sample at 10 mM yielding the value of background fluorescence F_{min}. F_{min} differs from F_0, as it does not include the initial level of fluorescence of NBD present in the sample. However, the value of F_0 could not be reliably obtained during analysis of ultracentrifugated samples.

In this experimental setup the total fusion efficiency is given by equation:

$$total\ fusion = \frac{F_{init} - F_{min}}{F_{max} - F_{min}} \cdot 100\%$$

And full fusion is given by equation:

$$full\ fusion = \frac{F_{full} - F_{min}}{F_{max} - F_{min}} \cdot 100\%$$

Additional comment on fluorescence measurement

While bR can act as a acceptor for NBD-PE fluorescence, due to overlap of their respective emission and absorbance spectra[2], the post fusion surface density of bR and NBD-PE should not significantly influence the measured intensity of NBD-PE, even for samples containing trimeric bR undergoing limited fusion (Table S3). If there was a bias present the degree of fusion would always be underestimated in the reported values.

Estimation of PEG coating on the surface of liposomes

A polymer bound surface has a Flory radius given by the equation[3]:

$$R_F = aN^{(\frac{3}{5})}$$

Where *a* is the PEG monomer size (3.8 Å)[4] and *N* is the degree of polymerization (on average 45.4 for 2K-PEG).
This yields R_F= 37.5 Å.
Additionally, for 2% of (18:1)2K-PEG-PE in DOPS based membrane the grafting density *D* (mean PEG-PEG distance) is equal to:

$$D = \sqrt{(\frac{A}{M_{PEG}})}$$

Where, *A* is the area per lipid and M is the mole fraction of PEG and M_{PEG} is the mole fraction of PEG (2%).
The area per lipid can be approximated as the fluid state area of DOPS, A_{DOPS}[5] 65.3 Å^2 giving a value of *D* = 57.1 Å.
As *D* is bigger than R_f, the PEG molecules will be present in a "mushroom" conformation with average area defined as a circle with *Rf* radius. In such a case PEG coverage of liposome will be given by:

$$C = \frac{\frac{1}{2}\pi R_f^2 \cdot M_{PEG}}{M_{PEG} \cdot A_{2K-PEG-PE} + (1 - M_{PEG}) \cdot A_{DOPS}}$$

and equal to approx. 34%.

Coarse Grain (CG) simulations of an individual fusion event

CG Methods

All simulations used GROMACS 2019.2[6] and the CG Martini force field 2.0[7,8]. The CG models of a nanodisc containing a monomeric bacteriorhodopsin (bR) and a lipid bilayer, locally approximating the surface of a liposome, were built using the CHARMM-GUI server[9,10]. MSP1E3D1 nanodiscs (with bR) were built to reflect the "delivery" lipid composition (lipid composition 1, Table S1) i.e. 85/15 ratio of DOPS/DOPC lipids, distributed symmetrically over the two leaflets. The "target" DOPS (lipid composition 2, Table S1) planar lipid bilayer was similarly prepared using the same tool. The structures were initially solvated with water and neutralized by Na^+ counterions. Next, they were energy minimized using the steepest descent gradient algorithm and subsequently equilibrated in five steps for 1 ns using increasing time steps of 2 fs, 5 fs, 10 fs, 15 fs and 20 fs.

After equilibration, the nanodisc, and the lipid bilayer structures were dehydrated and desalted. Next, the nanodisc was placed on top of the lipid bilayer using the GROMACS tools 'gmx editconf' and the distance between the nanodisc and membrane set to ~1.6 nm and the simulation box was resized to fit both the nanodisc and membrane. The whole system was solvated with water and neutralized by Na^+ counterions. Following this, 700 mM $CaCl_2$ was added to the system. While in the experimental setting the concentrations of positive cations used for induction of fusion did not to exceed 10 mM, in our simulation, to fully saturate the PS and PC headgroups, a concentration of 700 mM was used. This is in good agreement with the concentrations of Ca^{2+} experimentally found to be bound to the surface of PS/PC membranes which have been established to have a binding constant of 650 M^{-1} being able to bind more than one cation per headgroup exceeding simple coulombic interactions[11].

The whole system was subsequently energy minimized for 5000 steps using the steepest descent algorithm. Next, lipid headgroups in the z-direction and protein atoms were position restrained. The system was then equilibrated for 400 ns, using a timestep of 5 fs, allowing for the ion content to equilibrate. During a subsequent 10 µs production run no stalks initiating the fusion process were spontaneously formed. To initiate a stalk, after equilibration, a pulling potential was added to eight lipids of the nanodisc chosen randomly at the center of the nanodisc. Using this pulling potential, nanodisc and bilayer were pulled together at the center of the bilayer for 10 ns using a 5 fs timestep and this initiated the stalks. From this initial stalk state, upon removal of the pulling potential, five production runs for 10 µs were carried out using a timestep of 10 fs.

Default settings for the Martini forcefield were used, including use of a Verlet cut-off scheme with a 1.1 nm cut-off for both the coulombic (reaction-field) and van der Waals interactions[12]. We used v-rescale for the thermostat at 300 K, coupling the protein, lipids and solvent in separate groups. Equilibration was performed using the Berendsen barostat[13], using semi-isotropic coupling. For the production runs, the pressure coupling was switched to Parrinello-Rahman[14].

The box dimensions of the equilibrated system were 25.89 x 25.89 x 26.50 nm with a total of 148558 CG beads. The total system composition was as follows: 2052 lipids are present in DOPS planar lipid bilayer, MSP1E3D1 is composed of 280 lipids (42 DOPC and 238 DOPS), two proteins (bacteriorhodopsin and membrane scaffold protein), 7533 calcium ions, 2305 sodium ions, 15066 chloride ions and 84046 water beads (each bead representing four water molecules) are present. 10001 anti-freeze particles were also added to prevent the water from

freezing. A snapshot of the prepared starting setup, after the equilibration phase, is shown in Fig. S7A. To model a cylindrical pore on the DOPS lipid bilayer we applied cylindrical repelling force to the lipid tails in all three direction (x,y and z). This created a water pore with a radius of ~2.3 nm allowing lipid and water molecules movement[15].

CG Results: Radial stalk expansion leads to hemifused state with inverted micelle

In the original 10 µs production run, no stalks initiating the fusion process were spontaneously formed in presence of divalent ions. As proven by our experimental work, this is strictly a consequence of limitations of the CG modelling setup, most likely connected to insufficient dehydration of the membranes on the accessible time scale of the simulation[16]. To proceed further we introduced lipid contacts (known to be the first barrier in the fusion pathway) by forcing tail overlap between the lipids of the nanodisc and target membrane[17–19]. In the subsequent five independent production runs, this procedure resulted in spontaneous stalk formation within a 5 ns timescale (Fig. S7B).

Over the next 100-1500 ns, the stalk expanded rapidly as reported by the minimal distance between the phosphate groups of proximal leaflets dropping from ~1.5 nm to ~0.5 nm (Fig. S7C) and number of contacts between them increasing (Fig. S7D). This approach and expansion can be partially attributed to formation of *trans* Ca^{2+} bridges (here the Ca^{2+} is represented by a generic divalent cation bead) connecting the PS headgroups of apposed membranes (Fig S7F) and allowing for subsequent flip-flop of lipids from one membrane to another (Fig S7G), as evidenced by the increase in the number of tail-tail contacts (Fig. S7E).

The stalks expanded linearly and curved around the nanodisc until both their ends met and connected trapping an inverted micelle in between the nanodisc and lipid bilayer (Fig. S8A, B). The inverted micelle remained stable for the next 10 µs of the production runs trapping the nanodisc in the hemifused state. This entrapment is visible as the plateaus in distances between nanodisc and lipid bilayer and in the number of contacts between their proximal leaflets (Fig. S7C, D).

Notably, the minimal distance between the distal leaflet lipids of the nanodisc and the lipid bilayer (Fig. S9A) in one of the simulations (SIM2, colored red) decreased from ~6.5 nm to ~3 nm after 5 µs. The cause of this decrease was found to be the opening of the MSP monomer in contact with the lipid bilayer (chain A) (Fig. S9B) which in turn allowed for more lipids of the nanodisc to mix with the lipid bilayer. This opening of MSP provided a hint towards a possible fusion pathway, which was explored later.

Averaged across the replica simulations, we observed that only ~105 lipids of the nanodisc flip into the lipid bilayer but only into the nanodiscs proximal leaflet, which would correspond to ~35% (105/280) of lipid mixing and 0% full fusion. As this value is lower than one observed experimentally, we decided to try to force the progression of the fusion in the system further.

To test whether the progress of fusion was artificially hindered by the increasing accumulation of lipids entering the proximal leaflet of the lipid bilayer, we induced a pore in the membrane to allow flipflop of lipids to relieve the stress. The water pore was created by applying a cylindrical potential on the carbon tails of the lipid bilayer, had a diameter of ~2.3

nm and was located in the target membrane distal to the nanodisc. Artificial water pores are often introduced for equilibrating lipid vesicles[15,20] where they help lipids to move from one leaflet to another and initiate system relaxation[9]. Note that in the experimental setup, the amount of stress induced in the target membrane is much smaller, as the nanodisc size is small compared to the liposomal target.

The five replicate systems with the inclusion of a water pore were each simulated for an additional 8 µs. In all the five simulations, we observed the expected flip-flop of lipids between the proximal and distal membrane leaflets, however distal leaflet lipids of the nanodisc remained intact inside the membrane scaffold protein (Fig. S10A). Despite this treatment, in all cases, the nanodisc (with bR) remained hemifused to the lipid bilayer and the inverted micelle remained stable (Fig. S10B).

CG Results: Unwrapping MSP allows fusion to proceed

As the pore formation did not help in proceeding with the fusion, we decided to focus on the opening of the MSP, observed during Simulation 2 (Fig. S9B). To test whether presence of the MSP hinders full fusion, we removed both MSPs (as a proxy for MSPs opening and being removed from the fusion site) and made sure the system was electrically neutral. At this stage the cylindrical potential required for the pore formation was also removed in all the five systems. Next, we continued the simulations for another 5 µs. Immediately after removing the MSP, the distal leaflet lipids of the nanodiscs coalesced with the lipid bilayer (Fig. S11A). Nevertheless, the system remained trapped in the lens-shaped state (Fig. S11B) where the inverted micelle is surrounded by a flat membrane. Bacteriorhodopsin (bR) was found to span from the top leaflet of the lipid bilayer into the inverted micelle. This arrangement remained stable throughout the 5 µs simulation.

CG Results: Rupturing the inverted micelle to deliver bR

To force the completion of the fusion process we decided to rupture the trapped inverted micelle. To achieve this, lipids forming the inner layer of the micelle were removed, the system as a whole was subsequently neutralized (Fig. S12A) and then simulated for 100 ns. Within 10 ns a transient pore was spontaneously formed in the bottom leaflet, allowing the escape of the water trapped in-between the lipid bilayer. In four out of five simulations, this resulted in bacteriorhodopsin being adsorbed into the membrane with final topology being typical for a planar lipid bilayer (Fig. S12B).

CG Discussion

While the CG MD approach is inherently limited in reproducing the experimentally studied fusion events, as it is restricted to sampling events occurring on the nanosecond to microsecond timescale, it does point towards key events required for individual nanodisc-liposome fusion. The summary of the proposed fusion mechanism is presented in Fig.S13.

The initial stages of fusion are expected to proceed via the route established for the liposomes: first, the calcium ions overcome repulsion of the negatively charged PS headgroups[21] (Fig.S13 panel I); next, the membrane dehydration and curvature changes[22] lead to some form of a transient state (Fig.S13 panel II). Our results suggests that in this state an inverted micelle, caused by the linear expansion of the stalk, is formed and the MSPs still can be retained constraining lipids originally present in nanodisc. This type of stalk expansion is not specific to membrane protein loaded nanodiscs, having been previously reported for simulations of SNARE mediated nanodisc – membrane[23] and membrane – membrane fusion[24], as well as for the protein-free membrane fusion[25]. This was additionally confirmed in our simulation omitting bR where the fusion pathway remained the same (Fig. S14).

In our CG setup the formed inverted micelle proved to be stable enough not to allow the fusion to proceed, even when the tension introduced by asymmetric number of lipids in leaflets was removed using an artificial fusion pore and when the MSPs were subsequently removed. The inverted micelle, however is expected to be a transient lipid arrangement[26] and our experimental data shows that this entrapped state (Fig. S8) is not the final product of the fusion as in this state only ~35% lipid mixing (estimated by counting lipids released from the nanodisc) and no full fusion would be observed. Additionally, the rupturing of the inverted micelle (Fig. S12) is necessary to achieve the delivery of the membrane protein in membrane spanning fashion which was observed in experiments with OmpG (Fig.4,5).

In our simulations, the fusion of the distal nanodiscs leaflet only occurs after the removal of the MSPs (Fig.S10, S11). This suggests that opening of MSPs is required for the lipid mixing to proceed to the extent we observe in the experiments. ~84% of lipids are moved from nanodiscs to post-fusion liposomes (Fig. S17A; Table S5). Using this value and assuming fusion of both nanodisc leaflets with complete lipid randomisation the expected surface density of Rhod-PE in the resulting vesicles is ~0.17 mol% which corresponds to lipid mixing of ~73% (based on calibration curve; Fig. S1). The measured lipid mixing of ~78% in post-fusion liposomes for MSP1E3D1/monomeric bR nanodiscs (Table S4) thus confirms that fusion of both leaflets is occurring. This implies that the MSP present in the post-fusion liposomes does not retain its original tight, belt-like arrangement around the membrane protein and is expected to adapt a more open conformation on the liposome surface. The postulated opening is reasonable given that ApoAI and proteins derived from it can adapt to various curvatures[27], assume wide-open conformations[28,29] and diffuse in lipid bilayers upon incorporation into them[30]. Notably, given this requirement for flexibility, covalently circularised nanodiscs[31,32] might be not suitable for use in nano-CRAFT.

By removing the MSPs and the inverted micelle we reached a result where the membrane protein was delivered into the membrane and which aligned well with the experimental data.

This simulated fusion pathway is expected to apply only to events where the membrane protein is successfully delivered. As lipid transfer has been shown to be more efficient than the protein transfer (Table S5) a portion of MSPs (and bR carried inside nanodiscs formed by those) is expected to be removed from liposomes post-fusion. This differentiates the calcium driven mechanism from the SNARE based one, where the presence of the fusogenic proteins can stabilise the typically unstable fusion pore, not allowing for membrane coalescence nor the release of the MSP proteins[24,33]. Here, in the calcium driven fusion, the MSP removal is most likely occurring in the form of protein-lipid particles which after the lipid exchange retain mostly non fluorescent lipids. This pathway would be similar to detachment of ApoAI from

membranes observed in AFM studies[30] or even the ApoAI removal from chylomicrons[34]. The particles formed in events of this type experimentally are found in the higher sucrose fractions after ultracentrifugation confirming their nature as protein-lipid aggregates or reshaped nanodiscs rather than buoyant liposomes (Figure 3, S17). Importantly, the removal of MSPs after the delivery of bR is also possible and might explain why the transfer of membrane protein is slightly higher from one measured for MSP (Table S5).

Concerning the sequence of events the bursting of the micelle can happen either before the release of MSP (Fig.S13 panel III(a)), after it (Fig.S13 panel IV(b)) or concurrently with it. The last being the most probable scenario and a possible explanation for underestimation of full fusion in the MD simulations where the events were artificially sequential. With prompt and simultaneous micelle bursting and MSPs opening, more lipids might be transferred into the distal leaflet of the target membrane.

Lastly, the membrane protein in our simulation was delivered retaining the original orientation present in nanodiscs in reference to target bilayer (Fig. S12, S13 panel V). The absence of protein flipping suggests that if the orientation of nanodiscs can be controlled the delivery protocol could ensure desired orientation of the membrane protein in target bilayer.

Supplementary Data

Table S1 Compilation of lipid compositions used in this study

composition no.	description:	lipid constituent [mol%]					
		DOPS	DOPC	Rhod-PE	NBD-PE	PEG-2K-PE	Cy5-PE
0	liposomes for NBD-PE fluorescence correction	98	0	0	2	0	0
1	Delivery liposomes/nanodiscs, unlabelled	75	25	0	0	0	0
1*	delivery liposomes/nanodiscs, labelled	75	21	2	2	0	0
2	Target SUV liposomes, unlabelled	100	0	0	0	0	0
2*	target SUV liposomes, labelled	96	0	2	2	0	0
3	target PEGylated SUV liposomes, unlabelled	98	0	0	0	2	0
3*	Target PEGylated SUV liposomes, labelled	94	0	2	2	2	0
4	delivery nanodiscs for NMP-bR tracking, labelled	75	23	2	0	0	0
5	target GUV liposomes	75	23.5	0	0	0	1.5
5Ø	target not-fusable GUV liposomes	0	98.5	0	0	0	1.5
6	delivery nanodiscs for GUV leakage experiment	75	24.5	0.5	0	0	0

Note: The base composition is always set as 75 mol% of DOPS and 25 – n mol% of DOPC, where n is the mol% of all the additional components, e.g., fluorescently labelled lipids and PEG-lipids. Lipid composition used in the calibration curve as shown in Figure S1 were omitted for clarity.

Table S2 Compilation of lipid composition combinations used in experiments in this study

Experiment description:	compositions:	results presented in: Figures:	Supplementary figures:
fusion of delivery composition to target composition	1*+2	1B, 2E, 2F	
cross-fusion of delivery composition	1*+1	1C	
cross-fusion of delivery composition (DLS)	1*	1D, 1E	S2, S3
cross-fusion of target composition	2*+2	2E	
cross-fusion of target PEGylated composition	3*+3	2E	
cross-fusion of delivery target (DLS, NS-TEM, Cryo-EM)	3*	2A	
fusion of delivery composition to target PEGylated composition	1*+3	2B, 2D, 2E, 2F, 3A, 3B, 4	S16, S17, S18, S19, S20
NMP-bR and lipid delivery tracking	4+3	3C	
NMP-bR delivery tracking	1+3		S6
nanodiscs fusion with GUVs	1*+5		S23
nanodiscs fusion with GUVs control	1*+5Ø		S23
nanodiscs fusion with GUVs leakage (2-NBDG) experiment	6+5	5	S24
nanodiscs fusion with SUVs leakage (pyranine) experiment	1+5		S25

Note: lipid compositions are described in Table S1

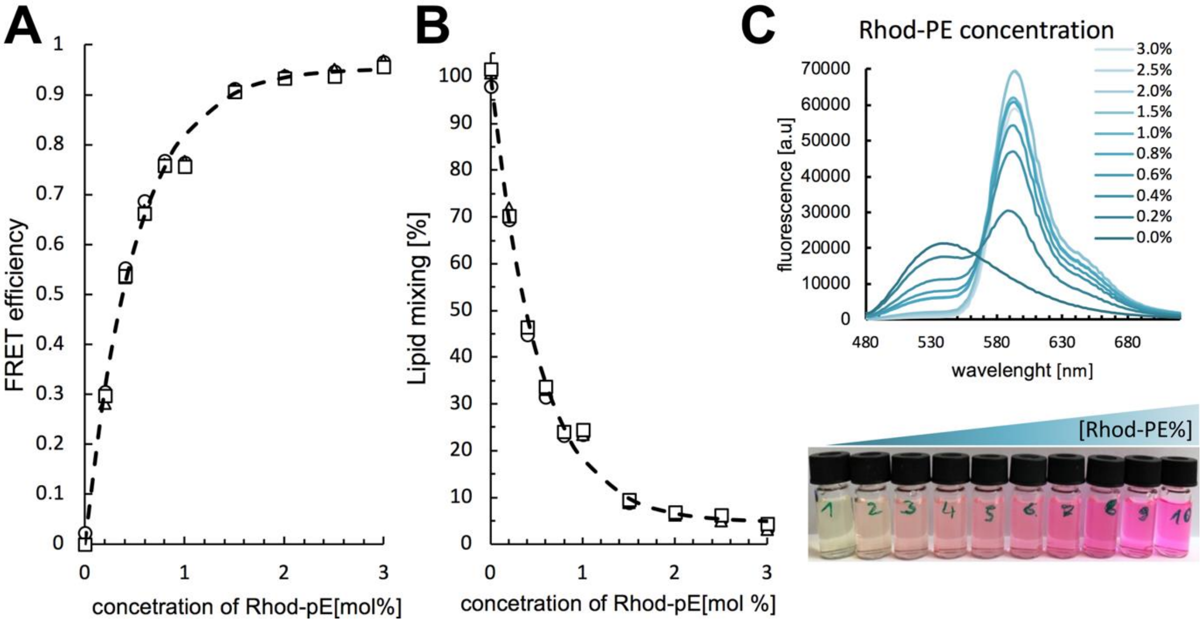

Figure S1 (A) FRET efficiencies with corresponding lipid mixing values (B) for different (surface) concentrations of the acceptor Rhod-PE (mol%) in pure DOPS 100 nm SUV liposomes with 1% of donor NBD-PE along with the representative fluorescence data and a photograph showing the appearance of the prepared samples (C). The curves were fitted to the average values of three technical replicates. The equation fitted for FRET efficiency was $y = 0.9539 \cdot (1 - e^{-1.9512x})$ and for Lipid mixing was $y = 0.9524 \cdot e^{-1.9469x} + 0.0460$.

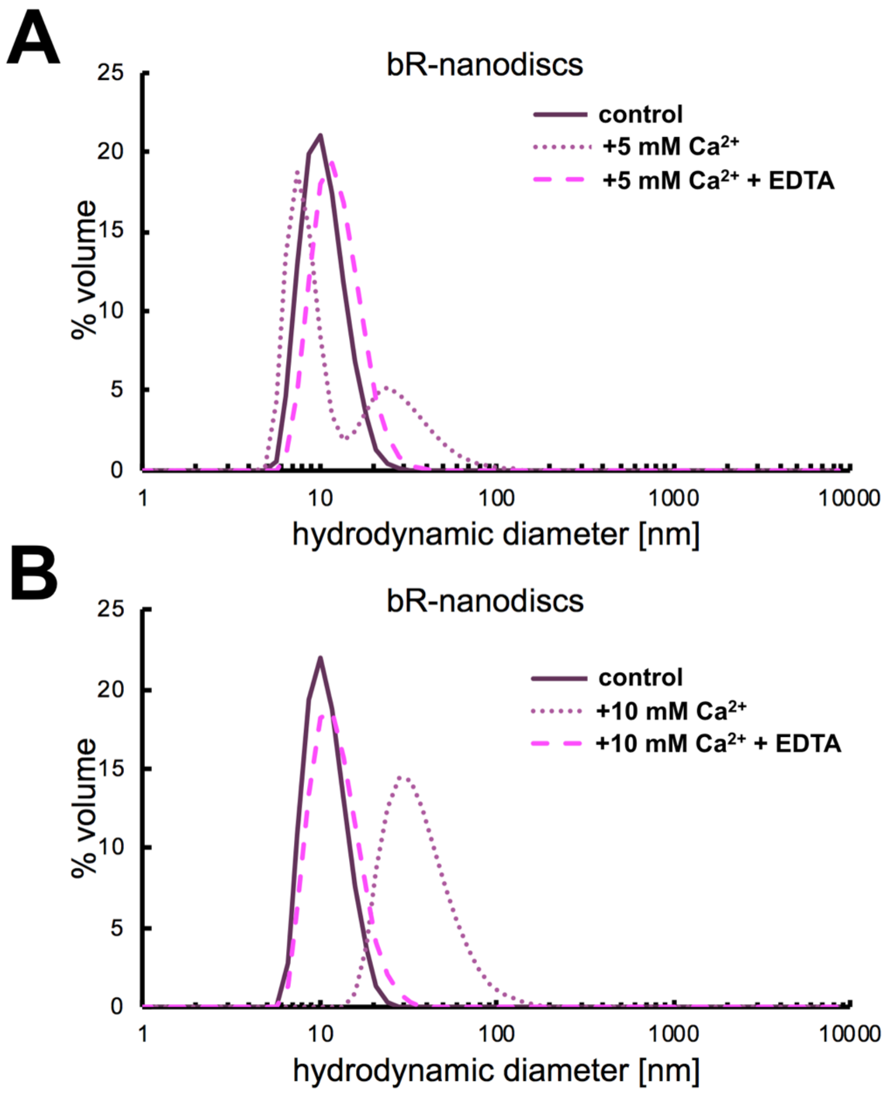

Figure S2 DLS measurements showing the aggregation of nanodiscs upon addition of 5mM (A) or 10 mM (B) of calcium and its subsequent reversal after chelation of the metal using EDTA.

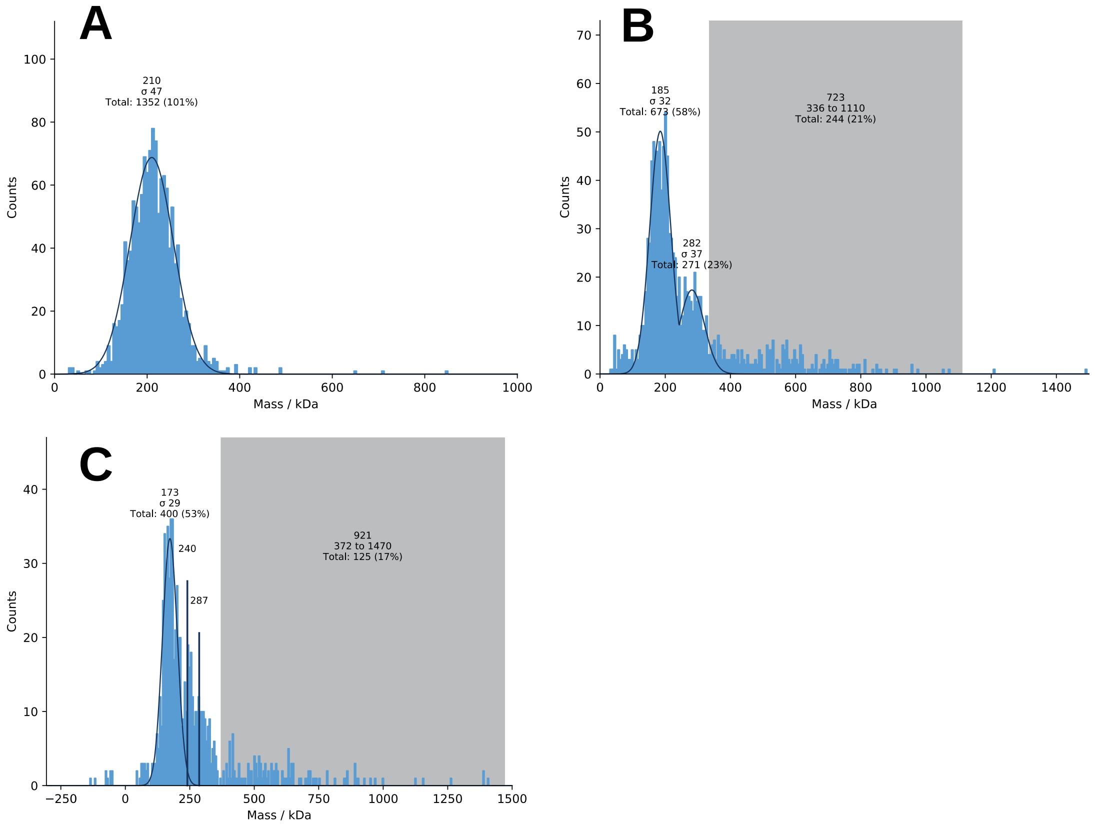

Figure S3 Mass photometry measurements showing the mass distributions of bR-loaded nanodiscs before fusion (A) upon addition of 10 mM calcium (B) and its subsequent reversal by chelation of the metal using 50 mM EDTA (C). Contrary to DLS the mass photometry results are not biased by the presence of larger particles, showing that the majority of bR loaded while undergoing size reshaping retain their compact size. The masses measured for the main peak show that prior to addition of Ca^{2+} the nanodiscs on average carry ~148 ± 58 lipids, 117 ± 40 in presence of calcium and 102± 36 after the chelation. The number of lipids were calculated after subtraction of the masses of two MSP1E3D1 protein and one bR protein assuming an average mass of lipid being the sum of 75% mass of DOPS and 25% mass of DOPC.

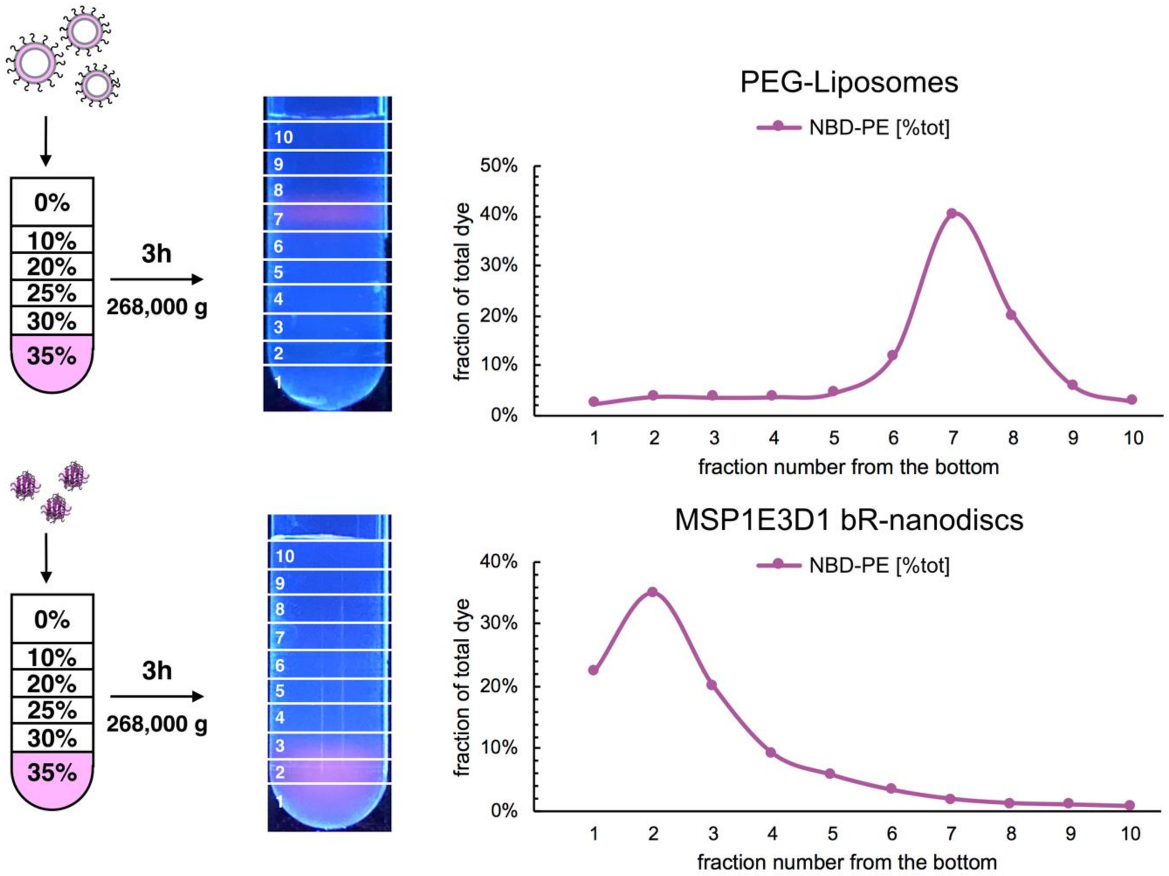

Figure S4. Ultracentrifugation of delivery composition (no.1*, see table S1) liposomes (top panels) and nanodiscs (bottom panels) in sucrose gradient. Schemes represent prepared sucrose gradients along with the 35% layer prepared by mixing the samples with a 70% sucrose solution. Images of ultracentrifuge tubes excited with broad UV showing the position of the dye with overlaid approximate position of collected fraction. The liposomes after the ultracentrifugation move to lower sucrose concentration layers, with no material remaining at the bottom of the tube, where the sample was loaded. The bR containing nanodiscs even after the ultracentrifugation, remain at the bottom of the tube. (left) Normalised fluorescence of NBD-PE for each fraction collected is shown as a fraction of total fluorescence measured for all fractions. The lines on the graphs are to guide the eye.

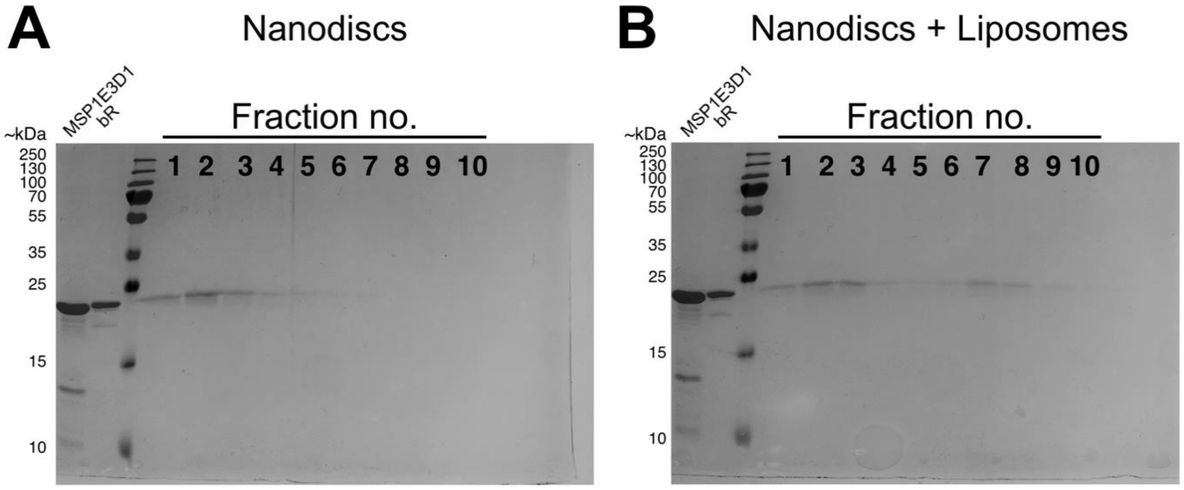

Figure S5 SDS-PAGE analysis of fractions collected for Nanodisc (A) and Nanodisc-Liposomes (B) after ultracentrifugation as shown and numbered in Figure 2 (main text).

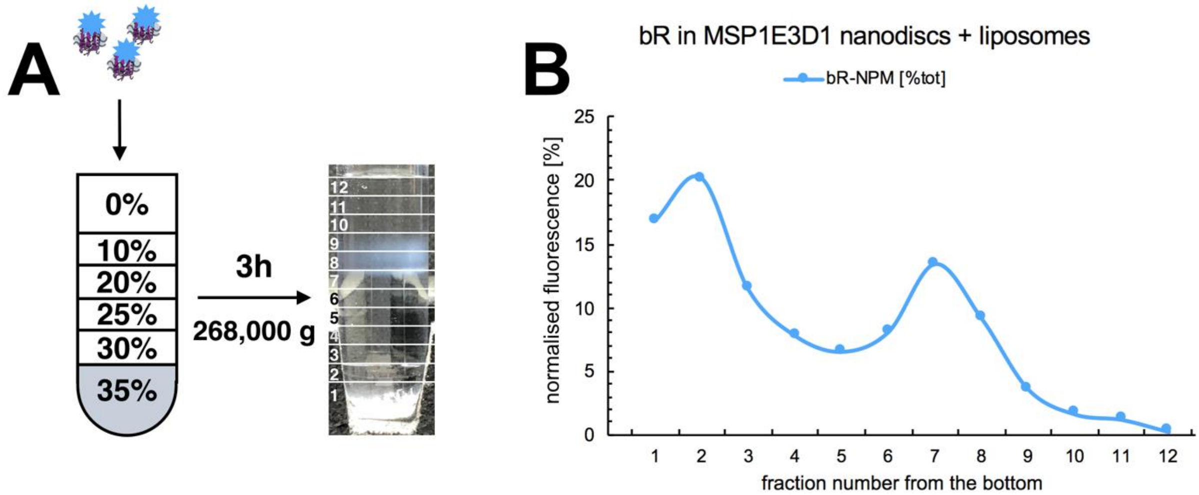

Figure S6 Ultracentrifugation analysis of fusion of NPM labelled bR in DOPS/PC 85/15 nanodiscs to DOPS/(18:1)-PEG2K-PE 98/2 liposomes. The liposomal fraction can be seen on the photograph of the tube as a band with increased light scattering (A). 385 nm fluorescence of NPM-labelled bR in collected fraction as a fraction of total fluorescence measured for all fractions (B). The lines on the graphs are not a fitted curve and are to guide the eye.

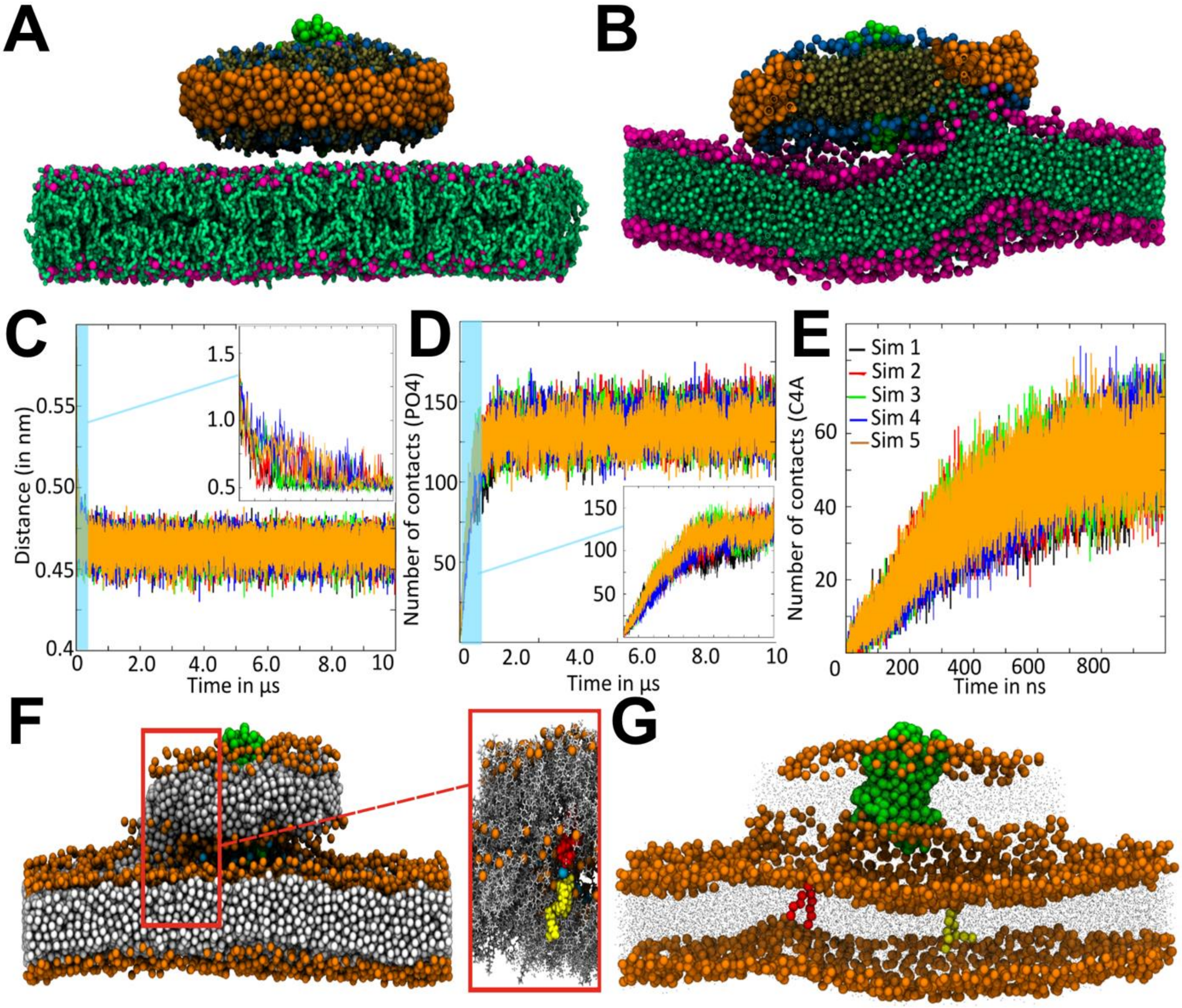

Figure S7 Initial stalk formation between nanodisc and lipid bilayer. (A) Original system setup shown with lipids in liquorice representation (atomic representation reverse mapped from CG) and the nanodisc with bR (VDW - CG representation) is placed in proximity to the lipid bilayer. (B) In the modified system a stalk was formed within 5 ns of the simulation. The headgroup of the nanodisc lipid is blue, and the tails are tan; the headgroup of the bilayer is magenta, and the tails are light green; bacteriorhodopsin is green, and MSP is orange. (C) Minimum distance between phosphate groups of the proximal leaflets of nanodisc and the bilayer stabilizes at ~0.47 nm. (D) Contacts between proximal leaflet lipids plateauing at ~ 120 to 145. (E) Contacts between the tails of the nanodisc and lipid bilayer confirms the mixing of lipid bilayer. (F) Calcium ion (cyan colour) promotes interaction between lipids in opposite bilayers (red/yellow). Atomistic representation reverse mapped from CG is shown in the inset. (G) Two lipids (red/yellow) flipped into the lipid bilayer after initial contact with the calcium ion. The membrane scaffold protein is removed in panels 1F and G for clarity, and the lipid headgroup is colored orange, the lipid tails are colored white, and the lipid tails in panel G are changed to dotted representation.

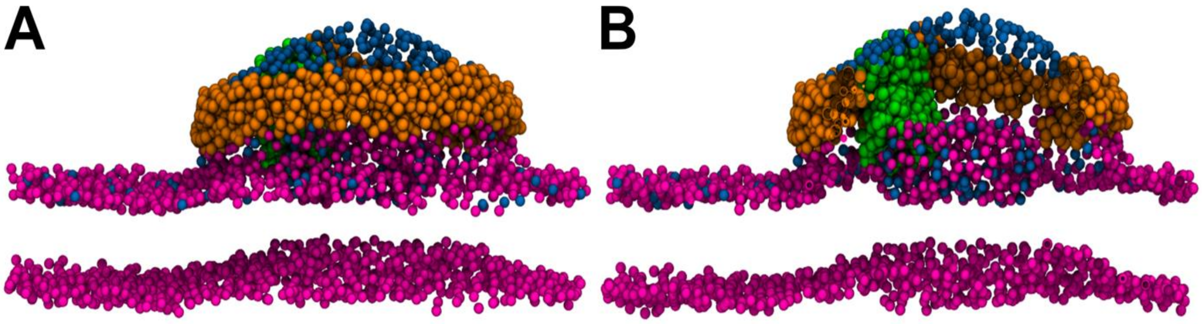

Figure S8 Inverted micelle formation due to stalk elongation. (A) Nanodisc (with bR) hemifused with the lipid bilayer. (B) Cross section of the system shows the micelle trapped in between nanodisc and lipid bilayer. In this figure, only the phosphate headgroups of the lipids (CG beads) are shown for clarity. The nanodisc lipid headgroup is blue, the bilayer headgroup is magenta, bacteriorhodopsin is green, and MSP is orange.

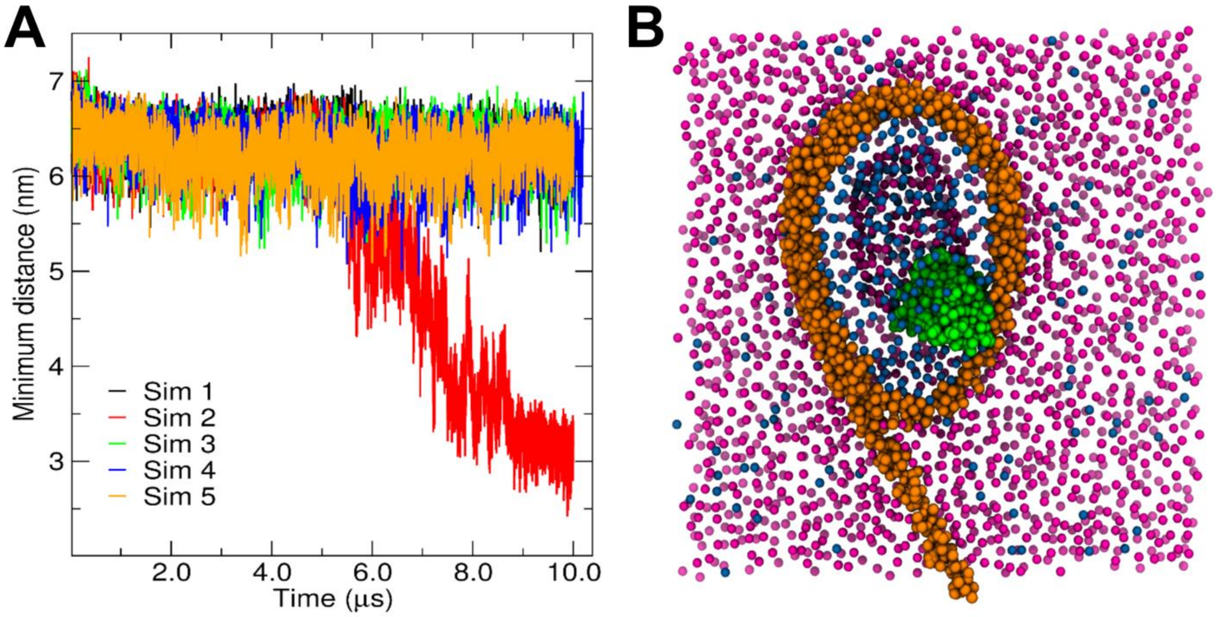

Figure S9 Unwrapping of MSP from the nanodisc. (A) Minimum distance between phosphate groups of the distal leaflets of nanodisc and the bilayer. (B) Chain A of the Membrane Scaffold Protein (MSP) opened during 10 μs simulation and this allowed more lipids to mix and decreased the distance between the distal phosphate groups in Sim 2. The colour scheme is identical to that shown in Figure S8.

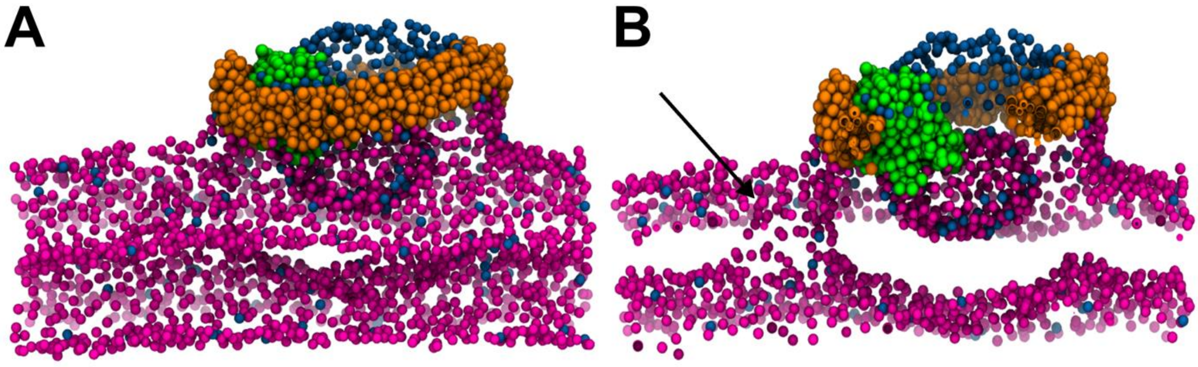

Figure S10 Effect of water pore on stability of hemifused state. Snapshots from one of the five replicates after simulating the system for 8 µs. (A) Nanodisc (with bR) is still hemifused to the lipid bilayer and outer leaflet lipids of the nanodisc remains intact while the proximal lipids mix (B) Cross section of the system shows the micelle trapped in between nanodisc and lipid bilayer is stable; the position of introduced pore is marked with an arrow. The color scheme is identical to that shown in Figure S8.

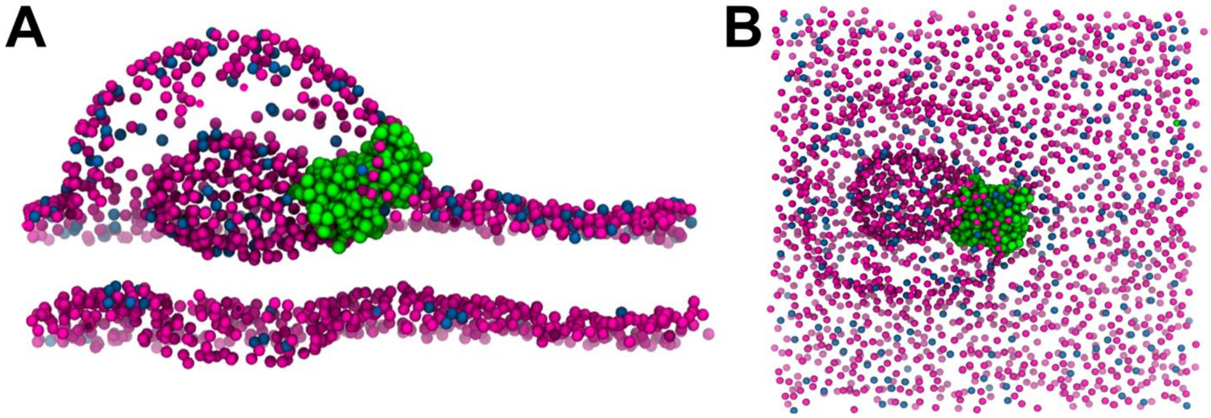

Figure S11 Removal of the MSPs leads to a lens-like state. (A) Inverted micelle is stable and is preventing bacteriorhodopsin from flipping inside the bilayer. (B) Inverted micelle is surrounded by the flat membrane, resembling a lens from the top. The color scheme is identical to that shown in Figure S8.

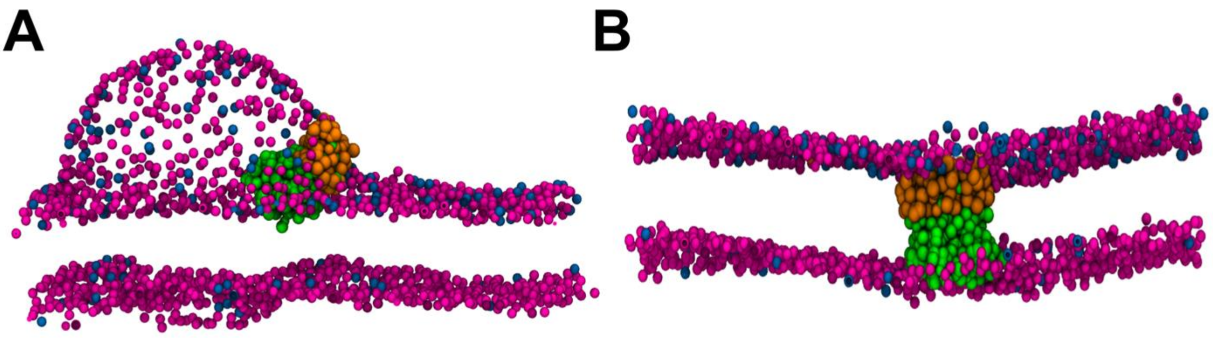

Figure S12 Delivery of bacteriorhodopsin via inverted micelle rupturing. (A) Inverted micelle disrupted and system simulated for 100 ns. (B) Bacteriorhodopsin was delivered to the lipid bilayer in the same orientation as it was in present in the nanodisc. The portion of bR originally positioned in the leaflet facing away from the bilayer is colored orange for clarity. Otherwise, the color scheme is identical to that shown in Figure S8.

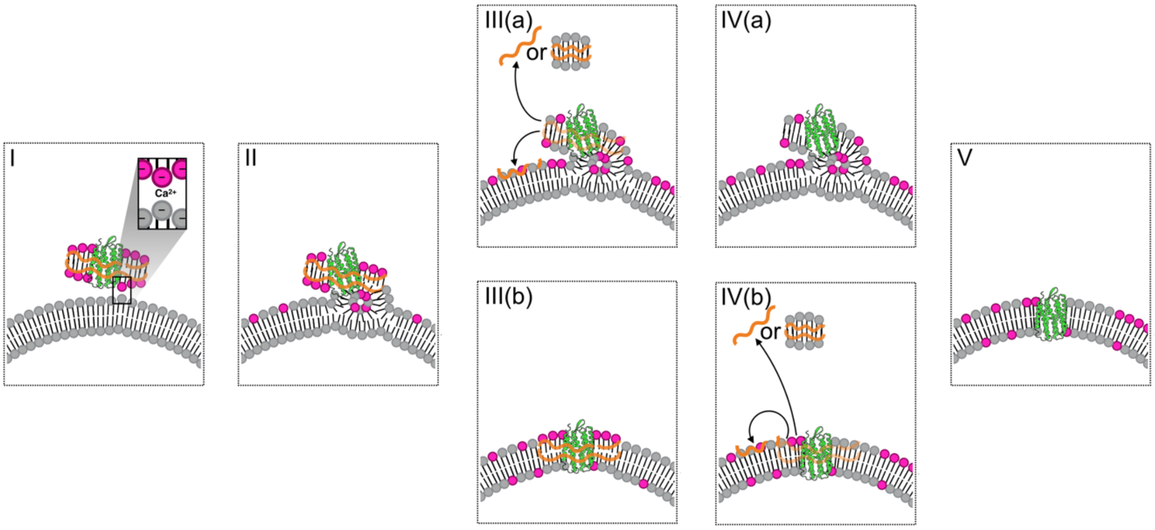

Figure S13 A scheme for potential fusion paths for individual nanodiscs. The screening of negative charge of apposed nanodisc and liposome membranes is facilitated by the presence of divalent Ca^{2+} cations (**I**). After the initiation of fusion via stalk formation, a metastable inverted micelle is trapped by linear stalk expansion (**II**). In one of the potential fusion paths first the MSP is released to the solution (lipid free or lipid bound) or to the surface of the liposomes (**III(a)**) and subsequently the freed micelle (**IV(a)**) is destabilized resulting in protein delivery and complete lipid mixing and delivery of lipids into the internal leaflet of the liposomes (**V**). In alternative path first the bursting of inverted micelle happens delivering the membrane protein and a portion of lipids into the internal leaflet of the liposomes (**III(b)**). This is followed by the release of MSP to the solution or the surface of the liposomes (**IV(b)**), allowing for completion of lipid mixing (**V**).

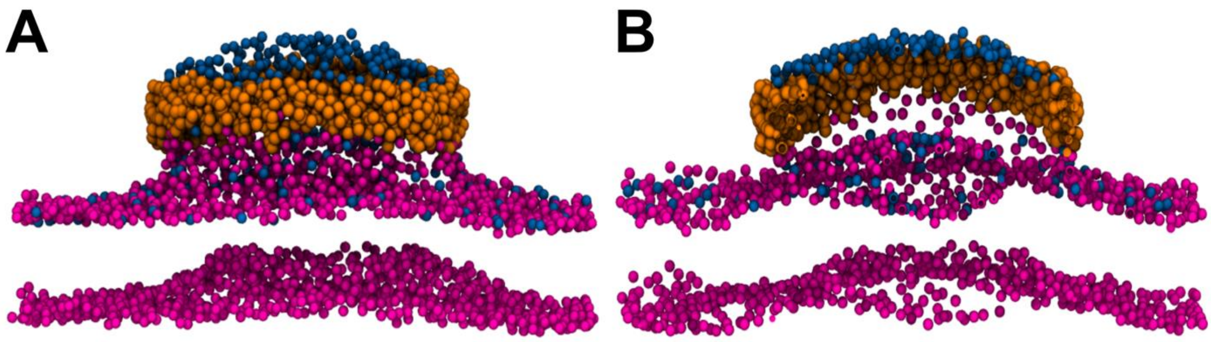

Figure S14 Simulation on MSP1E3D1 without inclusion of the bacteriorhodopsin in the nanodisc. The resulting structure traps an inverted micelle between the nanodisc and lipid bilayer (A) with the micelle clearly visible in the cross-section (B). The color scheme is identical to that shown in Figure S8.

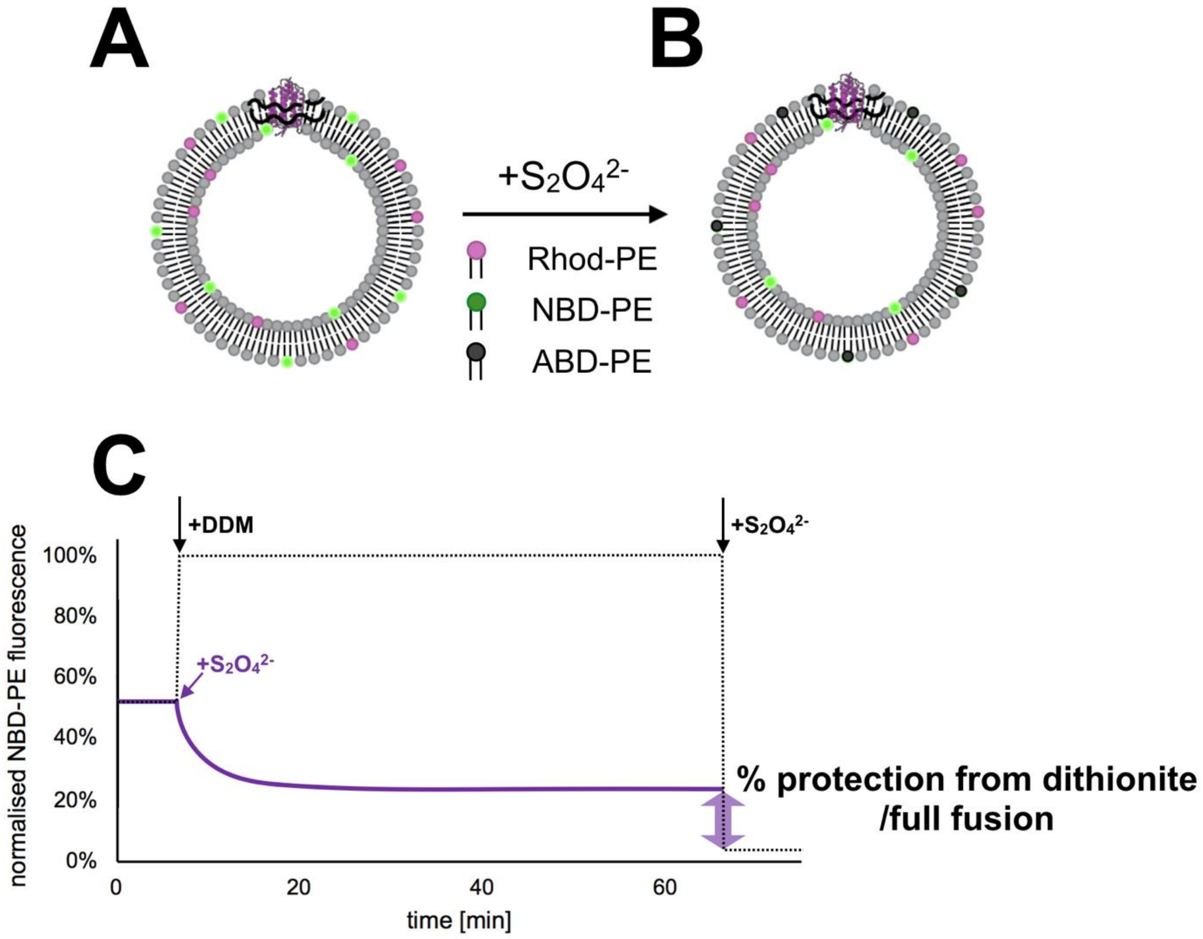

Figure S15 Scheme of full fusion assay. NBD-PE fluorescence at 530 nm is measured in post-fusion vesicles and after 5 minutes (A), membrane impermeable sodium dithionite ($S_2O_4^{2-}$) is added, irreversibility converting NBD-PE located in the outside leaflet of liposomes to nonfluorescent ABD-PE (B). The amount of 530 nm fluorescence signal remaining after this conversion corresponds to the percentage of lipids being transferred into the inner leaflet upon full fusion events. The 100% signal is established as the fluorescence measured for a duplicate sample dissolved using DDM and the background signal is established after sodium dithionite addition to the detergent dissolved sample (C).

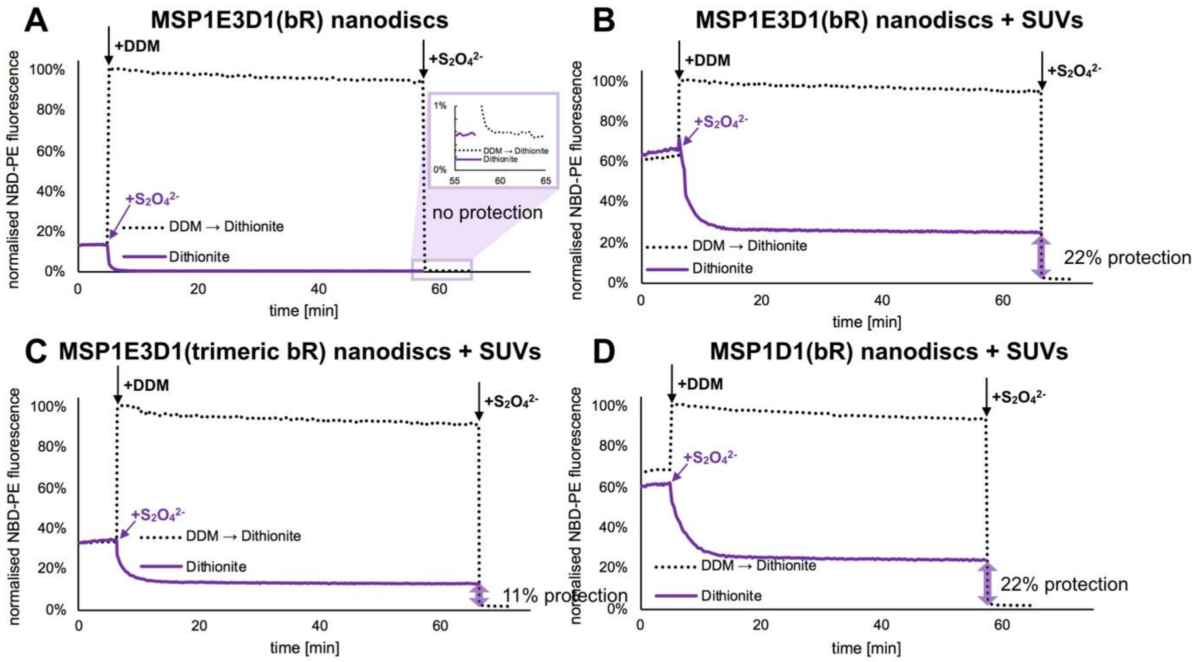

Figure S16 Dithionite quenching experiment for nanodisc samples prior to ultracentrifugation separation of post-fusion products. All of the samples underwent the fusion protocol as described in the methods. The times at which DDM and sodium dithionite ($S_2O_4^{2-}$) were added are indicated by arrows (A) MSP1E3D1 nanodisc bearing monomeric bR shows no protection from dithionite quenching, indicating that the all the lipids are readily accessible to solvent. Upon fusion of MSP1ED1 nanodiscs bearing monomeric bR (B), trimeric bR (C) and MSP1D1 nanodiscs bearing monomeric bR, protection from dithionite can be seen showing that a portion of lipids move to the interior of target liposomes. The values acquired for this experiment are presented in Table S3. The samples shown in panels A,B,C and D were subsequently used for analysis post ultracentrifugation and the corresponding results are shown in Fig.3 and Fig.S17.

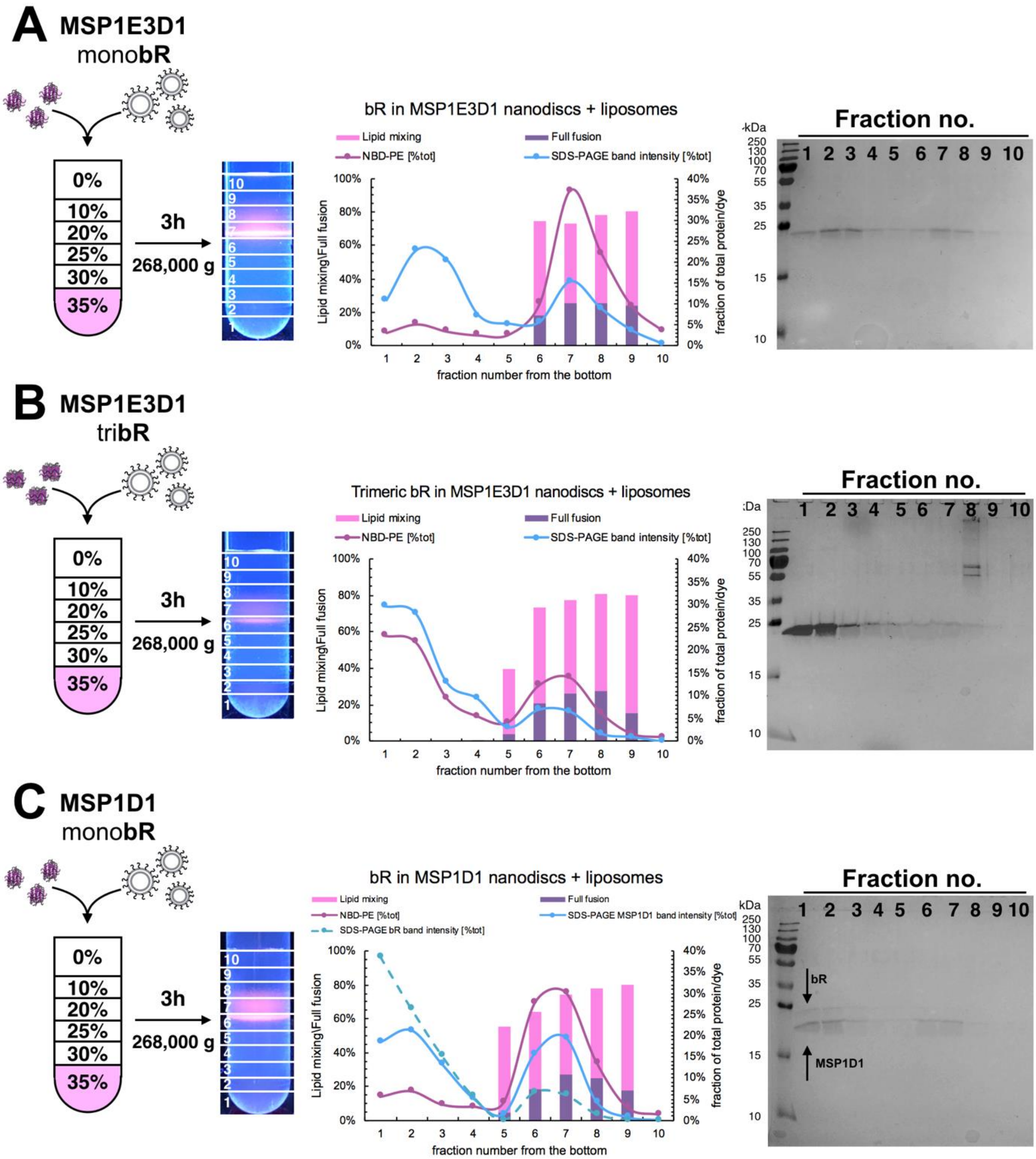

Figure S17 Compilation of analysis of three different fusion setups. The experimental schemes with the prepared gradients are shown. Photographs of tubes containing liposomes after fusion with (A) MSP1E3D1 nanodiscs with monomeric bR (mono**bR**), (B) MSP1E3D1 nanodiscs with trimeric bR (tri**bR**) and (C) MSP1D1 nanodiscs with bR. The approximate positions of fractions are overlaid. Measured % of lipid mixing and full fusion for corresponding fraction numbers (bars), normalized % of 530 nm fluorescence found in the given fraction and distribution of total protein found is the fraction as established by densitometric analysis of sub 25 kDa bands on SDS-PAGE gel is shown. Additionally, for MSP1D1 nanodiscs, % of total bR band intensity is shown (C). The lines on the graphs are not fitted curved and are presented to guide the eye. The results presented in (A) are an expansion of results shown in Fig.3A and are based on the same gel as in Fig.S5. Average values measured for fusion and full fusion for peak liposome fractions are compiled in Table S4. and the data for individual fraction measurements can be found in Fig.S18, S19 and S20. The densitometric traces can be found in Fig.S21, for simplicity for MSP1E3D1-based nanodiscs (A,B) only protein band of mass ~25kDa were used for analysis.

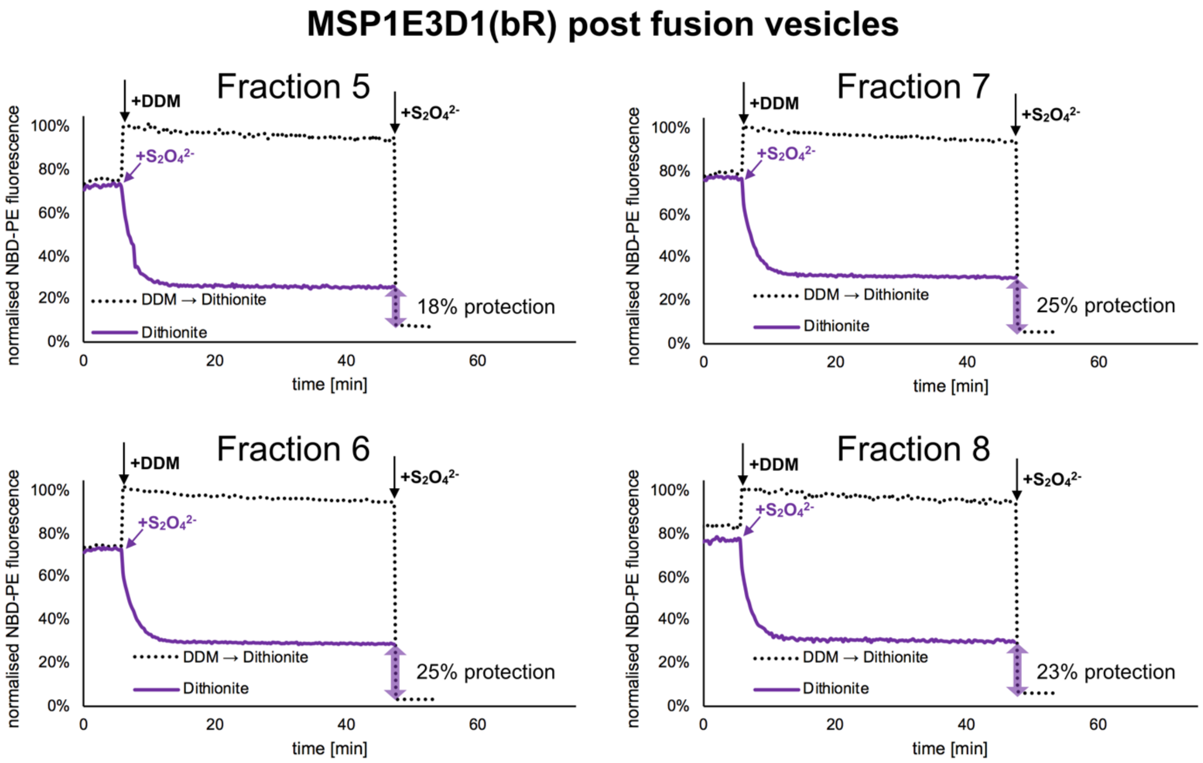

Figure S18 Dithionite quenching experiment for vesicular fractions collected after buoyancy based separation of products of fusion between MSP1E3D1 nanodiscs carrying monomeric bacteriorhodopsin (DOPS/DOPC/NBD-PE/Rhod-PE; 85/11/2/2) with SUV liposomes (DOPS/Pek-2K-PE; 98/2). The calculated amount of protection from dithionite (full fusion events) is shown on the graphs. The times at which DDM and sodium dithionite ($S_2O_4^{2-}$) were added are indicated by arrows. Weight averaged (based on %tot of NBD-PE fluorescence for the liposomal peak) for lipid mixing and full fusion are compiled in Table S4.

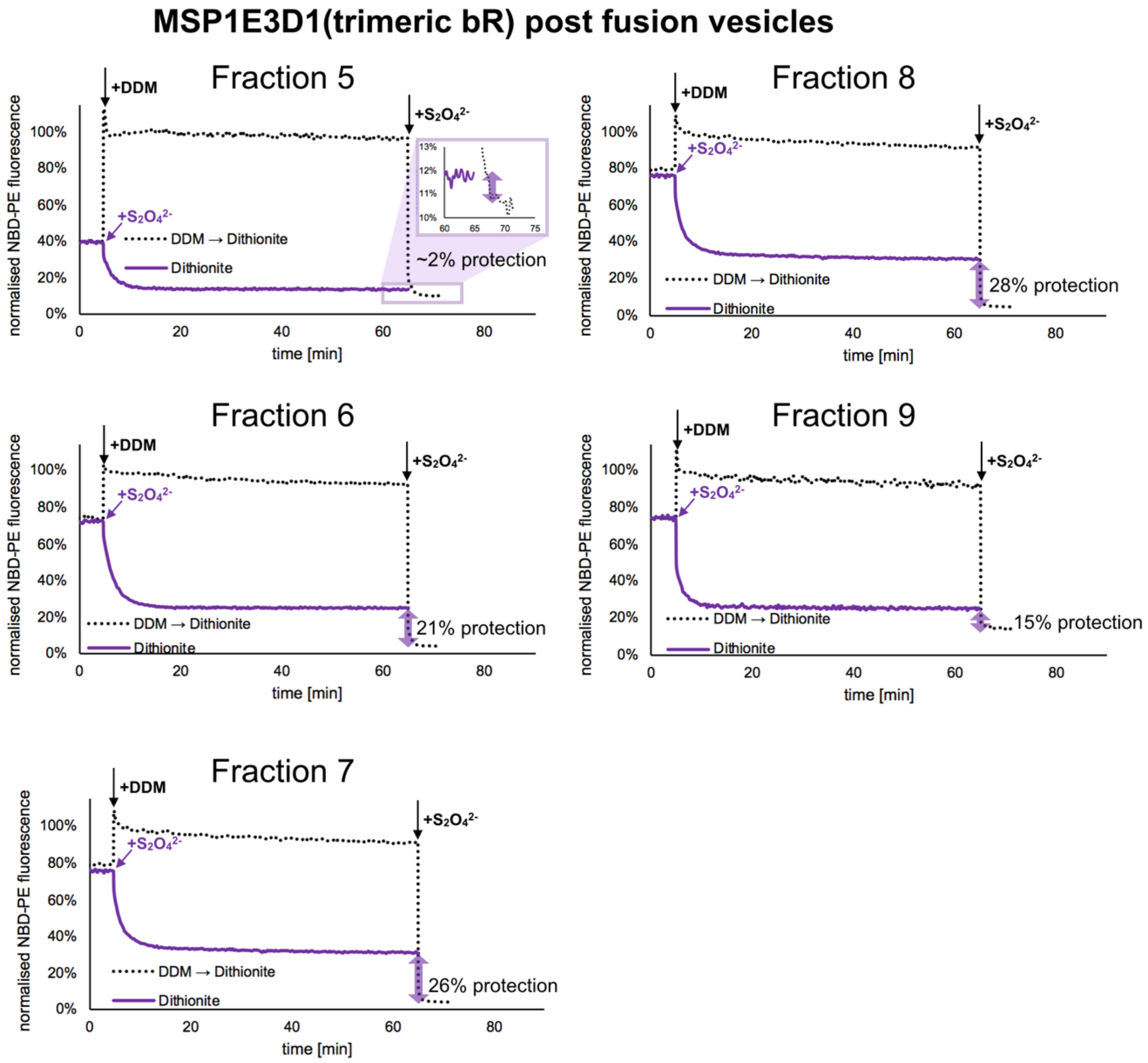

Figure S19 Dithionite quenching experiment for vesicular fractions collected after buoyancy based separation of products of fusion between MSP1E3D1 nanodiscs carrying trimeric bacteriorhodopsin (DOPS/DOPC/NBD-PE/Rhod-PE; 85/11/2/2) with SUV liposomes (DOPS/Pek-2K-PE; 98/2). The calculated amount of protection from dithionite (full fusion events) is shown on the graphs. The times at which DDM and sodium dithionite ($S_2O_4^{2-}$) were added are indicated by arrows. Weight averaged (based on %tot of NBD-PE fluorescence for the liposomal peak) for lipid mixing and full fusion are compiled in Table S4.

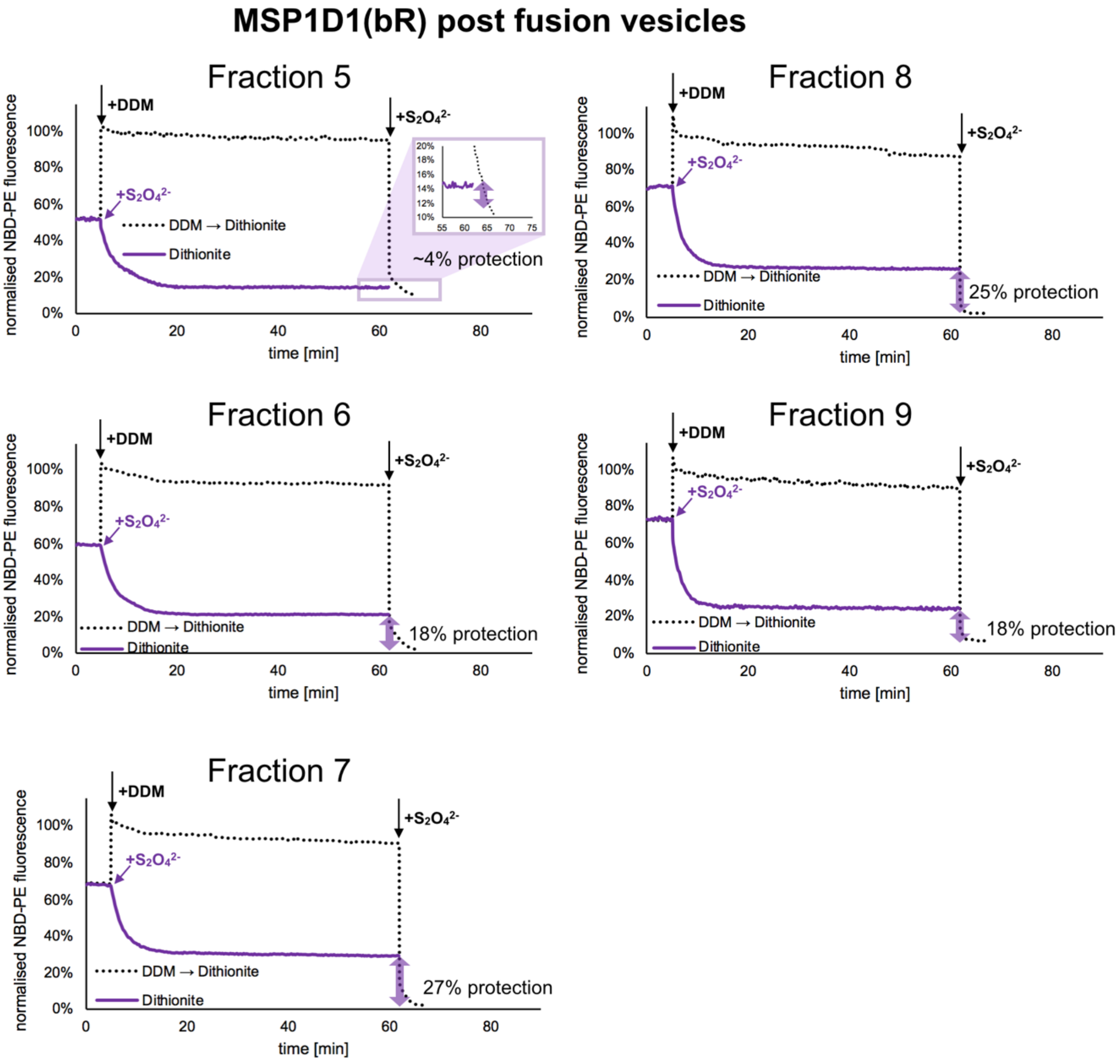

Figure S20 Dithionite quenching experiment for vesicular fractions collected after buoyancy based separation of products of fusion between MSP1D1 nanodiscs carrying monomeric bacteriorhodopsin (DOPS/DOPC/NBD-PE/Rhod-PE; 85/11/2/2) with SUV liposomes (DOPS/Pek-2K-PE; 98/2). The calculated amount of protection from dithionite (full fusion events) is shown on the graphs. The times at which DDM and sodium dithionite ($S_2O_4^{2-}$) were added are indicated by arrows. Weight averaged (based on %tot of NBD-PE fluorescence for the liposomal peak) for lipid mixing and full fusion are compiled in Table S4.

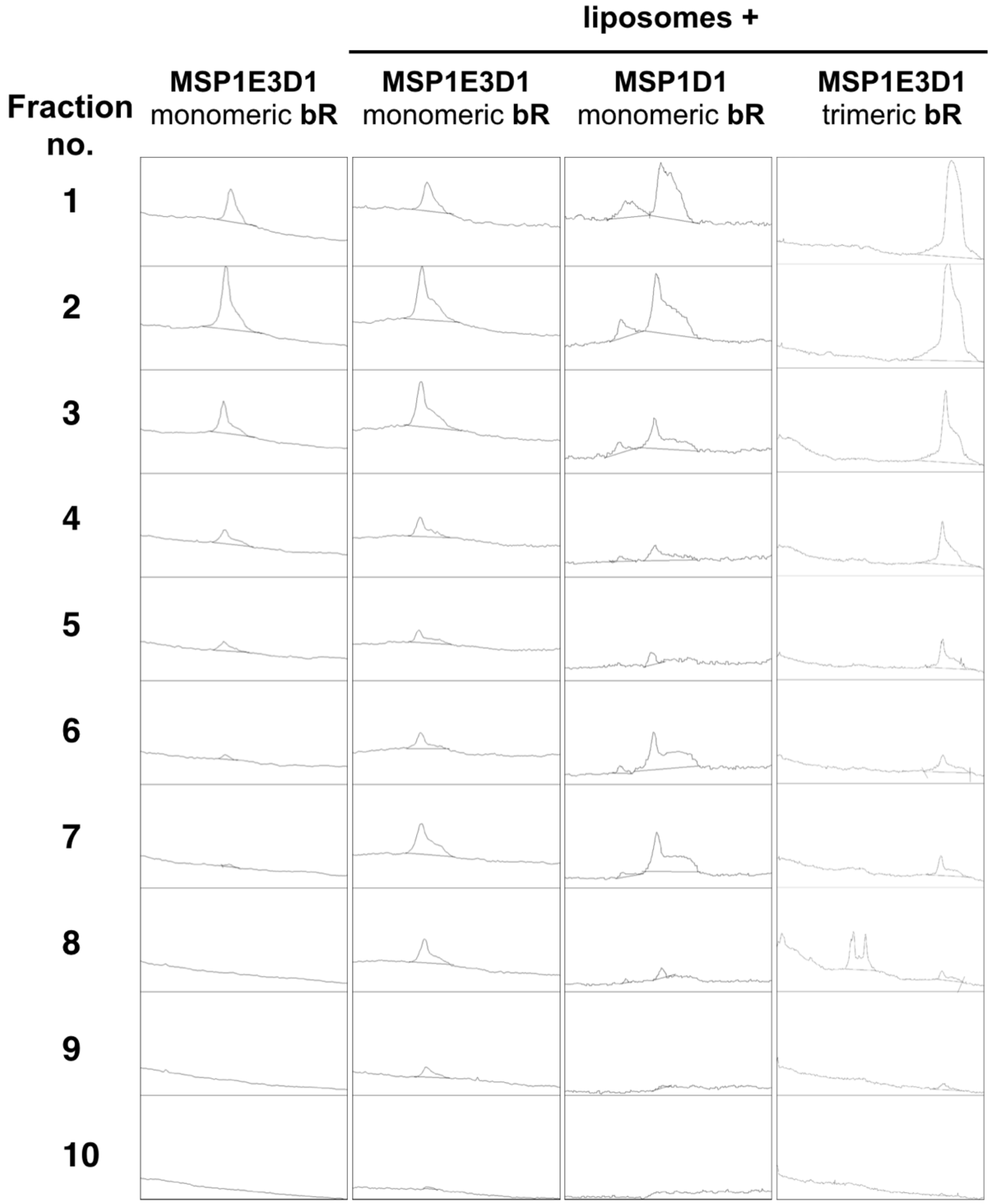

Figure S21 Densitometric traces of SDS-PAGE analysis of centrifugation fractions of nanodiscs alone and three studied types of nanodiscs after fusion with liposomes. The gels are presented in Fig. S5 and S17. The calculated values are presented on Fig. 3 and S17.

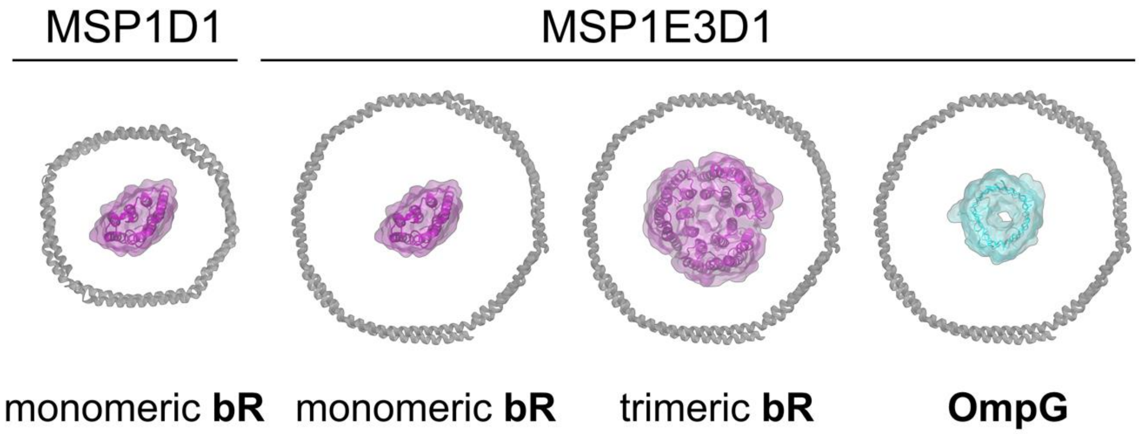

Figure S22 Overview of the studied nanodiscs variants. The MSP1D1 and MSP1E3D1 structures were generated as described by Stepien et. al[35]. The models were assembled in VMD software[36] using structure from pdb with following numbers: monomeric bR – 5BR5, trimeric br – 1BRR and OmpG – 2F1C.

Table S3 Compilation of the results for dithionite quenching experiments without the ultracentrifugation based separation of the products:

nanodisc type:	lipid mixing	protection from dithionite (full fusion)	full fusion/lipid mixing
MSP1E3D1 with bR	64.7%	22.0%	34.3%
MSP1E3D1 with trimeric bR	33.6%	10.8%	32.2%
MSP1D1 with bR	63.4%	22.9%	36.1%
MSPE3D1 with OmpG*	55.5%	2.1%	N/A

*Data for OmpG can be found in Fig.4B.

Table S4 Compilation of the results for dithionite quenching experiments post ultracentrifugation based separation of the products:

nanodisc type:	lipid mixing	protection from dithionite (full fusion)	full fusion/lipid mixing
MSP1E3D1 with bR	78.1%	24.5%	31.4%
MSP1E3D1 with trimeric bR	73.2%	21.9%	29.9%
MSP1D1 with bR	70.8%	21.9%	31.0%

Table S5 Compilation of results of lipid and protein transfer experiments

nanodisc type:	lipid transfer*	total protein transfer*	membrane protein transfer
MSP1E3D1 with bR	82.7–85.5%	37.2–41.3%	43.4–49.3%**
MSP1E3D1 with trimeric bR	35.5–39.7%	16.4–19.6%	N/A
MSP1D1 with bR	76.2–80.5%	27.2–27.9%***	14.1%*

*as quantified for fractions no. 5 or higher or 6 and higher, using fluorescent lipid content and SDS-PAGE band intensity; ** quantified by direct tracking of NPM-bR as seen in Fig.3C and Fig.S6; *** calculated for both MSP1D1 and bR SDS-page band intensities.

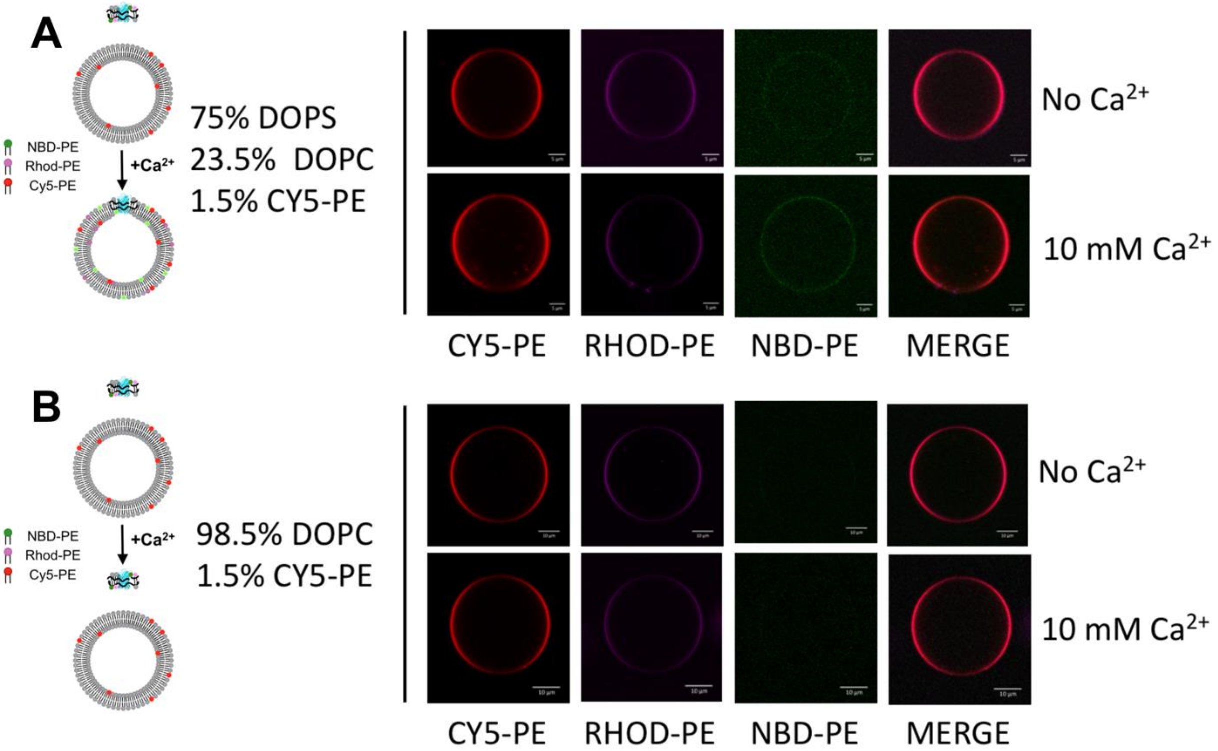

Figure S23 Confocal images of fusion between nanodiscs carrying OmpG and GUVs. The nanodiscs are labelled with Rhod-PE/NBD-PE fusion pair and after the fusion dequenching a faint signal from NBD-PE can be seen appearing on the periphery of the vesicle when DOPS based GUVs are used (A). The fusion however is not observed when the receiving GUVs do not contain DOPS (B).

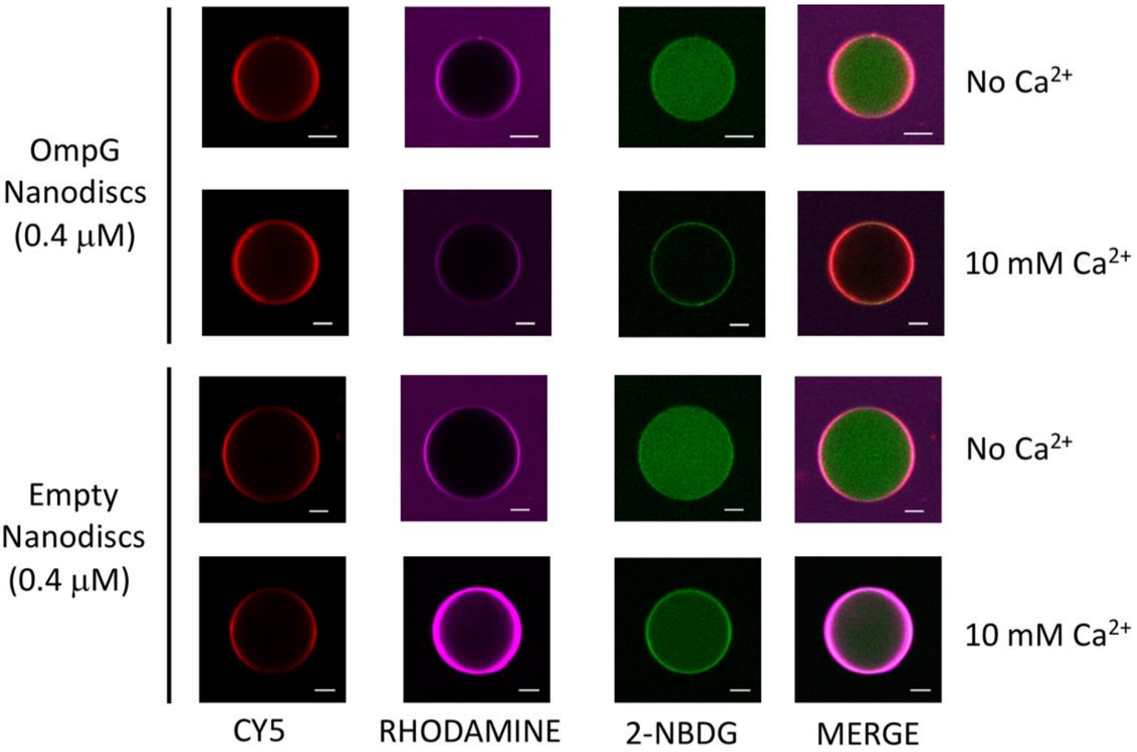

Figure S24 An independent repetition of the OmpG delivery induced leakage of 2-NBDG experiment described in Fig. 4 (main text). Bars denote 5 μm.

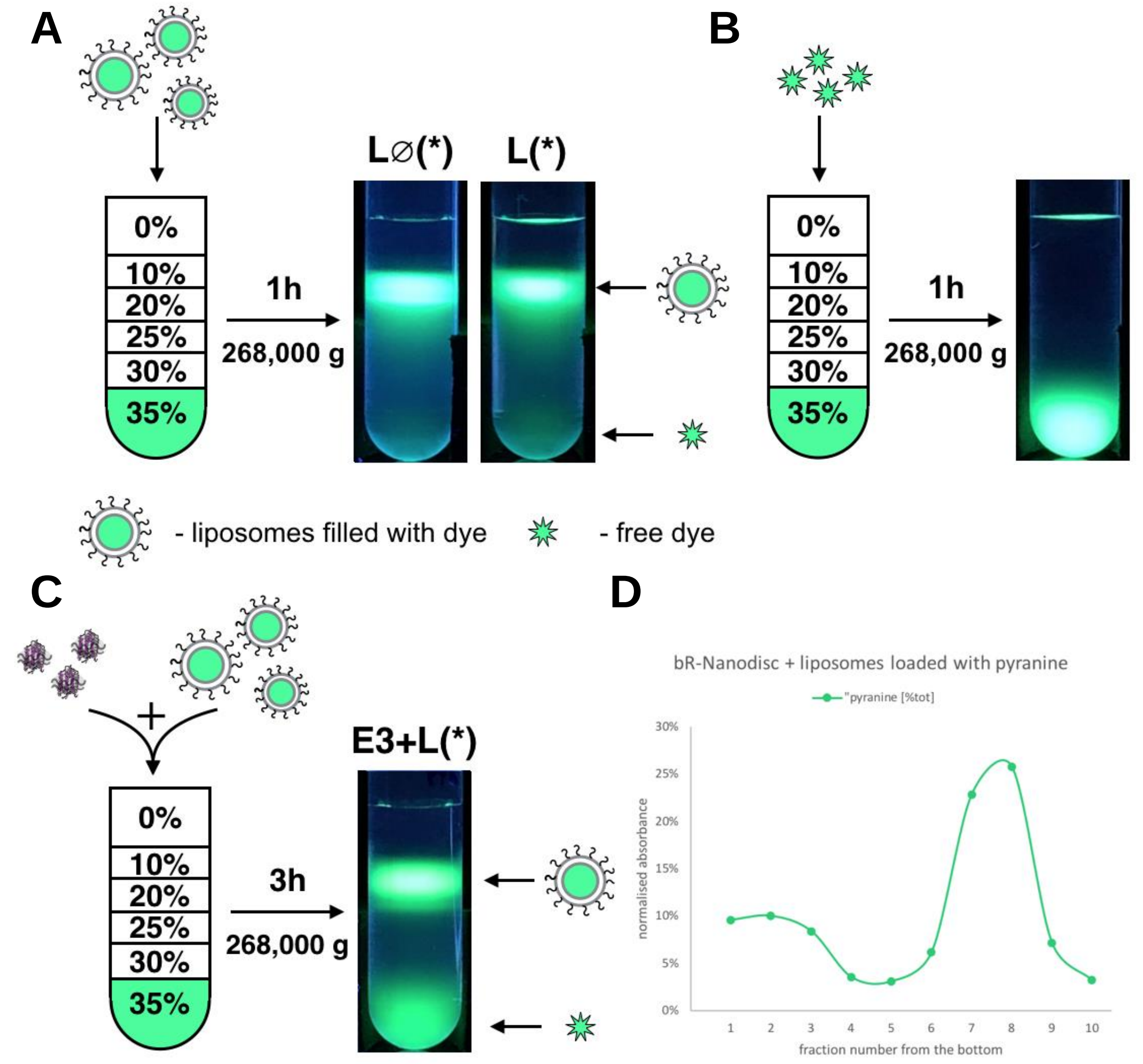

Figure S25 Leakage assessment of the fusion process involving SUVs. Photographs of tubes exited with broad UV showing (A) pyranine loaded in liposomes (LØ(*)), liposomes which underwent Ca^{2+} and EDTA/EGTA treatment (L(*)), (B) free pyranine and (C) sample after the fusion between DOPS/PC (85/15) E3 nanodiscs containing monomeric bR. (D) distribution of dye in fraction collected for the fusion sample. Measured % of pyranine concentration is given as normalized % of 464 nm absorbance found in the given fraction. The lines are presented to guide the eye. 65% of total signal was in the liposomal fraction (fraction 6-10) with 35% remaining in the fractions 1-5.

References

1. Struck, D. K., Hoekstra, D. & Pagano, R. E. Use of Resonance Energy Transfer To Monitor Membrane Fusion. *Biochemistry* **20**, 4093–4099 (1981).
2. Dumas, F., Sperotto, M. M., Lebrun, M. C., Tocanne, J. F. & Mouritsen, O. G. Molecular sorting of lipids by bacteriorhodopsin in dilauroylphosphatidylcholine/distearoylphosphatidylcholine lipid bilayers. *Biophys. J.* **73**, 1940–1953 (1997).
3. Kenworthy, A. K., Hristova, K., Needham, D. & McIntosh, T. J. Range and magnitude of the steric pressure between bilayers containing phospholipids with covalently attached poly(ethylene glycol). *Biophys. J.* **68**, 1921–1936 (1995).
4. Lee, H., Venable, R. M., MacKerell, A. D. & Pastor, R. W. Molecular dynamics studies of polyethylene oxide and polyethylene glycol: Hydrodynamic radius and shape anisotropy. *Biophys. J.* **95**, 1590–1599 (2008).
5. Petrache, H. I. *et al.* Structure and Fluctuations of Charged Phosphatidylserine Bilayers in the Absence of Salt. *Biophys. J.* **86**, 1574–1586 (2004).
6. Abraham, M. J. *et al.* Gromacs: High performance molecular simulations through multi-level parallelism from laptops to supercomputers. *SoftwareX* **1**–**2**, 19–25 (2015).
7. Marrink, S. J., Risselada, H. J., Yefimov, S., Tieleman, D. P. & De Vries, A. H. The MARTINI force field: Coarse grained model for biomolecular simulations. *J. Phys. Chem. B* **111**, 7812–7824 (2007).
8. Monticelli, L. *et al.* The MARTINI coarse-grained force field: Extension to proteins. *J. Chem. Theory Comput.* **4**, 819–834 (2008).
9. Qi, Y. *et al.* CHARMM-GUI Martini Maker for Coarse-Grained Simulations with the Martini Force Field. *J. Chem. Theory Comput.* **11**, 4486–4494 (2015).
10. Qi, Y., Lee, J., Klauda, J. B. & Im, W. CHARMM-GUI Nanodisc Builder for modeling and simulation of various nanodisc systems. *J. Comput. Chem.* **40**, 893–899 (2019).
11. Sinn, C. G., Antonietti, M. & Dimova, R. Binding of calcium to phosphatidylcholine-phosphatidylserine membranes. *Colloids Surfaces A Physicochem. Eng. Asp.* **282**–**283**, 410–419 (2006).
12. De Jong, D. H., Baoukina, S., Ingólfsson, H. I. & Marrink, S. J. Martini straight: Boosting performance using a shorter cutoff and GPUs. *Comput. Phys. Commun.* **199**, 1–7 (2016).
13. Berendsen, H. J. C., Postma, J. P. M., Van Gunsteren, W. F., Dinola, A. & Haak, J. R. Molecular dynamics with coupling to an external bath. *J. Chem. Phys.* **81**, 3684–3690 (1984).
14. Parrinello, M. & Rahman, A. Polymorphic transitions in single crystals: A new molecular dynamics method. *J. Appl. Phys.* **52**, 7182–7190 (1981).
15. Risselada, H. J., Mark, A. E. & Marrink, S. J. Application of mean field boundary potentials in simulations of lipid vesicles. *J. Phys. Chem. B* **112**, 7438–7447 (2008).
16. Leikin, S. L., Kozlov, M. M., Chernomordik, L. V., Markin, V. S. & Chizmadzhev, Y. A. Membrane fusion: Overcoming of the hydration barrier and local restructuring. *J. Theor. Biol.* **129**, 411–425 (1987).
17. Bruininks, B. M. H., Souza, P. C. T., Ingolfsson, H. & Marrink, S. J. A molecular view on the escape of lipoplexed dna from the endosome. *Elife* **9**, 1–16 (2020).
18. Smirnova, Y. G., Marrink, S. J., Lipowsky, R. & Knecht, V. Solvent-exposed tails as prestalk transition states for membrane fusion at low hydration. *J. Am. Chem. Soc.*

132, 6710–6718 (2010).
19. Pannuzzo, M., De Jong, D. H., Raudino, A. & Marrink, S. J. Simulation of polyethylene glycol and calcium-mediated membrane fusion. *J. Chem. Phys.* **140**, (2014).
20. Risselada, H. J. & Marrink, S. J. Curvature effects on lipid packing and dynamics in liposomes revealed by coarse grained molecular dynamics simulations. *Phys. Chem. Chem. Phys.* **11**, 2056 (2009).
21. Ohki, S. & Arnold, K. A mechanism for ion-induced lipid vesicle fusion. *Colloids Surfaces B Biointerfaces* **18**, 83–97 (2000).
22. Churchward, M. A. *et al.* Specific Lipids Supply Critical Negative Spontaneous Curvature—An Essential Component of Native Ca2+-Triggered Membrane Fusion. *Biophys. J.* **94**, 3976–3986 (2008).
23. Sharma, S. & Lindau, M. Molecular mechanism of fusion pore formation driven by the neuronal SNARE complex. *Proc. Natl. Acad. Sci. U. S. A.* **115**, 12751–12756 (2018).
24. Risselada, H. J., Kutzner, C. & Grubmüller, H. Caught in the Act: Visualization of SNARE-Mediated Fusion Events in Molecular Detail. *ChemBioChem* **12**, 1049–1055 (2011).
25. Jelger Risselada, H., Fuhrmans, M., Periole, X. & Marrink, S. The MARTINI Force Field. in *Coarse-Graining of Condensed Phase and Biomolecular Systems* 5–19 (CRC Press, 2008). doi:10.1201/9781420059564.ch2.
26. Siegel, D. P. Inverted micellar structures in bilayer membranes. Formation rates and half-lives. *Biophys. J.* **45**, 399–420 (1984).
27. Huang, R. *et al.* Apolipoprotein A-I structural organization in high-density lipoproteins isolated from human plasma. *Nat. Struct. Mol. Biol.* **18**, 416–423 (2011).
28. Nasr, M. L. *et al.* Covalently circularized nanodiscs for studying membrane proteins and viral entry. *Nat. Methods* **14**, 49–52 (2016).
29. Zhao, Z. *et al.* DNA-Corralled Nanodiscs for the Structural and Functional Characterization of Membrane Proteins and Viral Entry. *J. Am. Chem. Soc.* **140**, 10639–10643 (2018).
30. Plochberger, B. *et al.* HDL particles incorporate into lipid bilayers-a combined AFM and single molecule fluorescence microscopy study. *Sci. Rep.* **7**, 1–10 (2017).
31. Johansen, N. T. *et al.* Circularized and solubility-enhanced MSPs facilitate simple and high-yield production of stable nanodiscs for studies of membrane proteins in solution. *FEBS J.* **286**, 1734–1751 (2019).
32. Johansen, N. T. *et al.* Structural and Biophysical Properties of Supercharged and Circularized Nanodiscs. *Langmuir* **37**, 6681–6690 (2021).
33. Shi, L. *et al.* SNARE Proteins : One to Fuse and Fusion Pore Open. *Science (80-.).* **335**, 1355–1359 (2012).
34. Vigne, J. L. & Havel, R. J. Metabolism of apolipoprotein A-I of chylomicrons in rats and humans. *Can. J. Biochem.* **59**, 613–618 (1981).
35. Stepien, P. *et al.* Complexity of seemingly simple lipid nanodiscs. *Biochim. Biophys. Acta - Biomembr.* **1862**, 183420 (2020).
36. Humphrey, W., Dalke, A. and Schulten, K. VMD - Visual Molecular Dynamics. *J. Molec. Graph.* **14**, 33–38 (1996).
